# Analysis of Sources of Large Positioning Errors in Deterministic Fingerprinting

**DOI:** 10.3390/s17122736

**Published:** 2017-11-27

**Authors:** Joaquín Torres-Sospedra, Adriano Moreira

**Affiliations:** 1 Institute of New Imaging Technologies, Universitat Jaume I, 12071 Castellón de la Plana, Spain; 2 Algoritmi Research Centre, University of Minho, 4800-058 Guimarães, Portugal

**Keywords:** indoor positioning, Wi-Fi fingerprinting, simulation, positioning errors

## Abstract

Wi-Fi fingerprinting is widely used for indoor positioning and indoor navigation due to the ubiquity of wireless networks, high proliferation of Wi-Fi-enabled mobile devices, and its reasonable positioning accuracy. The assumption is that the position can be estimated based on the received signal strength intensity from multiple wireless access points at a given point. The positioning accuracy, within a few meters, enables the use of Wi-Fi fingerprinting in many different applications. However, it has been detected that the positioning error might be very large in a few cases, which might prevent its use in applications with high accuracy positioning requirements. Hybrid methods are the new trend in indoor positioning since they benefit from multiple diverse technologies (Wi-Fi, Bluetooth, and Inertial Sensors, among many others) and, therefore, they can provide a more robust positioning accuracy. In order to have an optimal combination of technologies, it is crucial to identify when large errors occur and prevent the use of extremely bad positioning estimations in hybrid algorithms. This paper investigates why large positioning errors occur in Wi-Fi fingerprinting and how to detect them by using the received signal strength intensities.

## 1. Introduction

It is well known that tracking, localization and navigation are interesting topics in academia and industry. They all have a common feature: the position of someone or something needs to be known. Positioning is valuable for end-users, tracking and advertising companies, government, and service providers, among many other involved actors. People can record their sporting activities and share them on social media [1]; companies can passively track people inside commercial areas [2,3] and analyse the customer patterns [4]; cars and robots can autonomously navigate and be tracked [5,6,7]. In recent years, location-based services (LBS) and real-time location systems (RTLS) have grown. The daily use of many smartphone applications, which require positioning, has been one reason for their growth. In fact, the LBS and RTLS market is expected to be USD 77.84 Billion by 2021 to support local search, recommender systems, e-commerce and business intelligence, among others.

This paper is focused on positioning [8], which is a term commonly associated to Global Navigation Satellite Systems (GNSS) such as GPS, Gallileo, GLONASS or BEIDU. However, there are some scenarios where GNSS cannot operate adequately and other solutions are required to support positioning. Indoor environments are of special interest since many studies show that people spend most of their time indoors [9,10,11,12], but GNSS signals do not penetrate structural elements and obstacles with ease. The GNSS solutions that work outdoors might not properly work indoors because radio signals can be easily blocked, attenuated or reflected [13].

In contrast to outdoor scenarios, there are many solutions and technologies to provide positioning indoors [14,15] and the deployment of a particular indoor positioning system depends on the operating area [16]. Crucial decisions about the base technologies and techniques are taken when systems are being designed and a trade-off among accuracy, costs, ubiquity, availability, scalability and other features is carefully considered [16]. Although accuracy is the key objective to be optimized, the priorities depend on the scenario. Autonomous robot navigation might require high precision approaches, whereas the positioning error may be less important in pedestrian-based applications. Thus, the requirements, the base technology and the deployment are different in both scenarios.

Wi-Fi fingerprinting is a well known indoor positioning technique, which consists of two stages: calibration (off-line phase) and operation (on-line phase) [17,18,19]. The former stage is related to the site survey where the received signal strength intensities (RSSI values) from multiple wireless access points (WAPs or simply APs) are recorded at predefined well-known positions. Each record is called a Wi-Fi fingerprint and it can be considered a snapshot of the Wi-Fi signal strength from the multiple APs received at a given point. A calibration database, or radio map, is generated with all the fingerprints collected at this first stage across the operational area. The latter stage, operation, is related to the positioning itself. A fingerprint is collected at an unknown point and a matching algorithm is applied to estimate its position. This matching algorithm estimates the position by using the known positions from the most similar fingerprints stored in the calibration database. The terms Wi-Fi, WiFi, 802.11 and WLAN are commonly used as synonyms in the literature.

**Figure 1:**
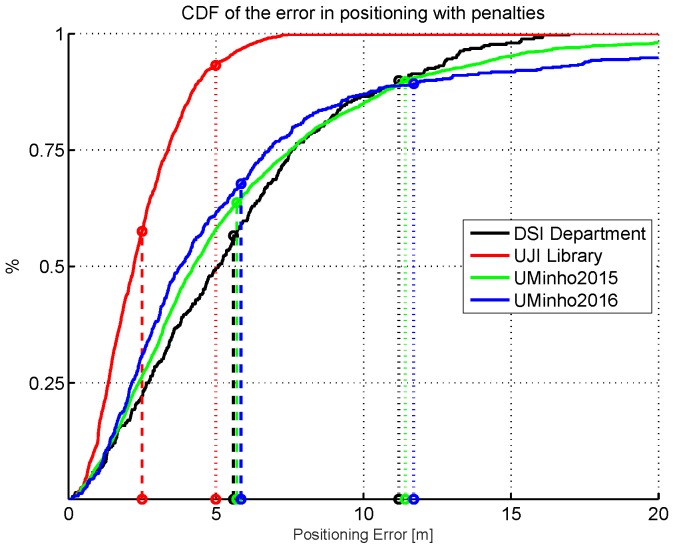
Cumulative distribution of the positioning error (CDF) for four different cases. Simple Wi-Fi fingerprinting system based on kNN at the DSI department (University of Minho, Portugal); Simple Wi-Fi fingerprinting system based on kNN at a small area of the university library building (Universiat Jaume I, Spain); UMinho system at the 2015 IPIN competition; UMinho system at the 2016 IPIN competition. The errors and penalties in floor detection are not considered in any of the results shown. Dashed vertical lines indicate the average error, whereas dotted vertical lines indicate twice the average error.

Wi-Fi fingerprinting assumes that all fingerprints, and therefore the corresponding RSSI values, are similar at a given point over time. Typical mean positioning errors are in the range of a few meters [20], which is enough for pedestrian indoor positioning and navigation in most of the cases. The observed mean error highly depends on the matching algorithm used and the scenario. Heterogeneity of hardware, density and distribution of calibration and operational points, density and distribution of APs, and the materials present in the environment, among many other factors, affect the mean positioning error. The consequence is that the same indoor positioning algorithm might report different accuracies in different areas [21]. However, unexpected and unacceptably large positioning errors are often observed in working systems deployed in real environments, even when the mean positioning error is low, as depicted in the examples shown in Figure 1. Figure 1 shows the Cumulative Distribution Function (CDF) for 1 kNN-based IPS using a database collected at the DSI department (Univeristy of Minho, Guimarães, Portugal), a Library area (Universitat Jaume I, Castellón, Spain) and the UMinho systems that participated in the 2015 and 2016 IPIN competitions [21,22]. In all cases, the percentage of operational fingerprints that are reporting a positioning error higher than twice the average error is significant (higher than 10% approx).

Although the vast majority of positioning errors are around the mean value, there are always a few cases whose positioning error is much larger, e.g., the UMinho IPS provided positioning errors higher than 20 m in the 2015 and 2016 IPIN competition (see Figure 1). While these large errors might be acceptable in some application areas, such as pedestrian navigation in shopping malls, other applications, such as robot navigation or tracking of vehicles for autonomous driving [23], are more demanding and do not tolerate these large errors.

In an attempt to leverage the attractive characteristics of fingerprinting while trying to circumvent its limited accuracy, some researchers have proposed hybrid positioning methods where fingerprinting is combined with other techniques. Examples of this approach are the combination of fingerprinting with pedestrian dead reckoning [24]; map matching and the physical structure of the buildings [25]; and Bluetooth [26]. Across these works, several combining techniques are also used, with the Kalman and Particle filters being the most frequent ones [27]. In this context, knowledge about the statistics of the errors enables the optimal combination of the position estimates provided by the merged solutions.

While the general trends of the statistics of errors in Wi-Fi fingerprinting are well known (e.g., see Figure 1), estimating the error associated with each individual position estimate is still a challenge and an open research topic. Of particular interest is the detection of cases where the errors are very large. So far, this research challenge has only been addressed by a few researchers, among them the works done by Depster et al. [28], Lemelson et al. [29], Farshad et al. [30], Zhi-An Deng [31] and Berkvens et al. [32].

Montgomery established that the nuisance factors in an experimental setup can be controlled, uncontrolled or, even, unknown [33]. This paper analyses how many controlled and uncontrolled factors have a direct impact on Wi-Fi fingerprinting accuracy: grid size; density and location of APs; density of fingerprints and positioning algorithm and its parameters. The “unknown” factor is also covered by assuming that the RSSI values (and, therefore, the noise present in the RSSI readings in a given position) follow a Gaussian distribution. Although this assumption is not always true, it significantly simplifies the computations with little performance difference according to [34]. In particular, the main objective of this paper is to explore why large positioning errors occur and how they can be detected by using only the information of the fingerprints, i.e., the RSSI values.

The rest of this paper is organized as follows. Section 2 reviews the related work on exploring and reducing the positioning error. Section 3 describes fingerprinting. Section 4 introduces the comprehensive study of the sources of (large) positioning errors in deterministic fingerprinting. Section 5 shows the relation observed between large errors and fingerprints and the results on real scenarios. Section 6 presents the conclusions drawn from this work.

## 2. Related Work

This section reviews the existing works that address the reduction of positioning errors in Wi-Fi fingerprinting.

Kaemarungsi and Krishnamurthy [35] developed a framework for analyzing a simple deterministic indoor positioning system based on the Euclidean distance to compare and match the fingerprints. The authors claimed that the accuracy of a positioning system could be determined, in terms of the probability of estimating the correct position, from the number of access points, grid spacing and path loss exponent. However, the analysis performed did not consider the accuracy and precision in the position estimate. Later, in 2012, they presented a statistical data analysis of the RSSI values in [36]. One of the main findings was that positioning errors were proportional to the increase of the standard deviation of RSSI values, which depended on the WLAN card and scenario.

Youssef and Agrawala [37] presented an analytical method in order to obtain the optimal strategy for selecting the user position. An analytical analysis was also performed to study the impact of averaging multiple fingerprints on accuracy. This work showed that the accuracy of their indoor positioning system was consistent under different user profiles when the number of visible access points was reasonable at each possible position. One of the main objectives of the work done by Youssef and Agrawala was to find the function that minimized the probability of positioning error at the operational stage.

Krishnakumar and Krishnan [38] linked the minimum uncertainty with a lower hit on the median error and they also made some important observations about the dependence of the estimation uncertainty and various factors: signal variance, number of APs, distance between the APs and the signal propagation constant.

Wallbaum [39] used some analytical models and the empirical multi-wall radio propagation model [40] to investigate the influence of the following main parameters on indoor positioning: RSS deviation, number of visible access points, grid granularity and AP geometry. This work presented a comparative study of analytical and experimental results, which showed that both captured the qualitative behaviour of the real system. Wallbaum concluded that it should be investigated whether the models actually represent the upper and lower bounds of the positioning error.

Dempster et al. [28] introduced an analysis of RSSI variance, where it was shown that the user’s orientation at a reference point influences the RSSI value captured by a device. Averaging fingerprints just for position (i.e., the measurements taken at the same reference point without considering the user’s orientation) had higher variance than averaging the fingerprints per position and orientation, so averaging by position and orientation was more discriminative. Moreover, this paper concluded that the relationship between the real and RSSI distances of two fingerprints was poor. Comparing fingerprints with a not totally well-suited distance metric was suggested to be one of the reasons for positioning error. Finally, the authors highlighted that the physical underpinning might be easily ignored in dBm-based fingerprinting, being the RSSI values wrongly treated as unitless numbers.

Lemelson et al. [29] proposed four algorithms for estimating the error with just the information from fingerprints and improved the overall accuracy of the IPS proposed by Haeberlen et al. [41].

1. Applying efficient fingerprint clustering and using the cluster area information as a measure of the error (the real position is in the cluster in 90% of cases);
2. Using Leaving One (Out) fingerprint to compute an averaged map with the positioning error at well-known positions;
3. Using the average distance among all the *k* best candidates at the operational stage (the *k* references fingerprints that are most similar to the operational one) as the error estimation;
4. Using the variance of RSSI measurements at the operational stage to estimate the position error.

The results showed that a combination of the four algorithms significantly improved the accuracy of the IPS in two scenarios. However, this work did not introduce any study or analytical model in order to understand the origins of the (large) positioning errors as had been done in previous works [35,37,38,39].

Farshan et al. [30] took a microscopic look at Wi-Fi fingerprinting using smartphones. They firstly introduced seven definitions to improve the matching process at the operational stage using a deterministic approach (e.g., Radar [17,42]) and a probabilistic-based approach (e.g., Horus [37,43]). Three different distance metrics were considered in the former approach and two well-known distributions for RSSI modelling were used in the latter. The results showed that the combination of fingerprint definition and estimation algorithm that yields the best positioning accuracy highly depends on the environment and even on the floor for a given large environment. Moreover, they analysed the impact of frequency band on fingerprinting. The RSSI is more stable for the 5 GHz band, which enables the IPS to provide better positioning accuracy. The 5 GHz band is less affected by temporal signal variations due to people movement because the probes are sent at a higher bit-rate than the 2.4 GHz band probes. Also, the low variability is also due to the low co-channel interference in the 5 GHz band. Finally, the presence of Virtual APs (physical devices emitting multiple Wi-Fi networks) also reduces the positioning error because the AP density increases, which seems to have a positive correlation with accuracy, and the temporal variability inherent to wireless signal propagation is better captured. All results were provided as CDFs but they were truncated to errors of up to 10 m (large errors were omitted).

Zhi-An Deng et al. [31] proposed an indoor positioning system that exploits the data from Wi-Fi, smartphone sensors, landmarks and user motion status. One crucial step in their system was to detect outliers in RSS values, i.e., operational fingerprints that might have attached a large positioning error. An extended Kalman filter fusion approach might be negatively affected by an estimation with a large positioning error which can propagate to future position estimations, degrading positioning accuracy. Zhi-an Deng et al. proposed using the averaged weight of the calibration points in the trusted area as an indication of RSS reliability.

Berkvens et al. [32] studied the uncertainty of a Wi-Fi fingerprinting positioning system by relating the positioning error with the conditional entropy in the position posterior probability distribution. The uncertainty is commonly calculated as the positioning error using samples with well-known ground truth. Berkvens et al. proposed using the conditional entropy of a posterior probability distribution as a complementary measure of uncertainty, which had the advantages of being dynamic and not requiring ground truth. Based on this, they proposed a sensor model where the conditional entropy is an indicator of the upper bound of the positioning error for a particular operational estimate, i.e., a low conditional entropy value means that the positioning error is low, whereas a high value means that the positioning error might be high.

In general, most of the papers that have dealt with knowing the origins of the positioning error have the objective of reducing the average positioning error or showing a few features that originate from the positioning error. This paper is focused on investigating where the (very) large errors occur; as far as we know, there is no previous paper with this main objective.

## 3. Wi-Fi Fingerprinting

Wi-Fi fingerprinting is a well-known indoor positioning solution which relies on a fundamental assumption: the Wi-Fi signals measured in the environment have a unique signature—the Wi-Fi fingerprint—at a given position.

Wi-Fi fingerprinting has two differentiated phases: calibration (or training) and operation. In the calibration phase, which occurs before the operational phase, the environment is surveyed in order to create a radio map (also known as calibration or reference dataset). This process is empirically done by collecting Wi-Fi fingerprints at different reference points whose positions are well known. Regarding this stage, there is no clear best strategy and slight differences and assumptions can be found in the already proposed systems. Later, during the operational phase, a new fingerprint is collected with the signal strength of all detected APs at an unknown position. This fingerprint is compared with the fingerprints in the radio map and the position is computed using the positions of the most similar reference fingerprints.

Wi-Fi technology was initially designed to support communications, not for positioning. The propagation of radio signals in an indoor environment is not easy to predict due to the presence of people [35,44] and existing obstacles, which create reflections, refractions and multipath interference [19] that impair very precise positioning. Close locations might share similar fingerprints and, therefore, the typical accuracy of Wi-Fi fingerprints is within a few meters (1–10 m according to [20]). Advanced fingerprint methods report an accuracy of about 1 to 4 m [14,45] but the heterogeneity of testing environments hinders a fair comparison. Even in those cases where the positioning error is low, there are large errors. For instance, the UMinho system reported an average error of 6.33 m in the 2016 IPIN Competition [21], but large errors were observed: in 5.7% of cases, the positioning error was higher than 20 m and in two cases the error was higher than 40 m.

## 4. Error Sources in Wi-Fi Fingerprinting

This section is devoted to investigating the origins of positioning errors in Wi-Fi fingerprinting by means of simulated data. The analyses performed in this paper are based on a real scenario, since our long-term objective is to minimize large positioning errors for a particular application in that scenario. However, we consider that most of our findings are useful and can be extrapolated for fingerprinting in general.

The target scenario corresponds to a large laboratory used for polymer research work (PIEP) at the University of Minho, where an indoor positioning system has to be deployed. The space is around 50 × 20 m wide and more than 8 m high and, in many aspects, is very similar to a factory plant, with large machinery, plenty of metal structures and tools, and some quite large open spaces. Therefore, the considered scenario for simulation is a rectangular area of 50 × 20 m that resembles the real laboratory. Also, an initial AP distribution with eight Access Points installed near the longer walls has been considered: four of them (APs 1 to 4) placed at 3.9 m above the floor, and the other four (APs 5 to 8) placed at 5.4 m as done in [46].

In the simulations, the default indoor positioning system base parameters are as follows: kNN algorithm with k=1 (or closest match); the above-mentioned distribution of APs; reference fingerprints in a 1 m grid; and 10 fingerprints per reference point. Simulated base data (RSSI values) are generated with the free space path loss model (see Equation (1)). The selected values for the IPS and the FSPL balance efforts —to generate the radio map and compute the operational fingerprints—, resemble some features of real environments, and they ease the study of the rest of the parameters.

(1) $$FSPL=20\cdot \log_{10}\left(d\right)+20\cdot \log_{10}\left(f\right)+adjustconstant$$

where *d* refers to the distance to the AP and *f* corresponds to the signal frequency.

### 4.1. Quantization of RSSI Values

**Figure 2:**
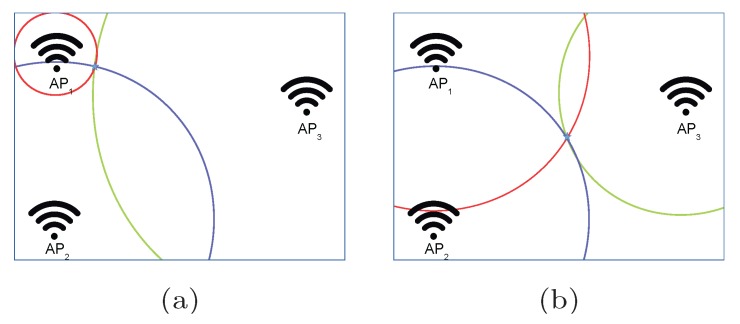
Illustrative examples of the areas where a fingerprint can be placed in the optimistic world. (**a**) Fingerprint placed near to $AP_{1}$. (**b**) Fingerprint equidistant to all APs.

The Wi-Fi signal is an electromagnetic wave whose intensity is attenuated as it propagates through space. In the optimal case, where the signal is not affected by any external interference, the free space path loss model (see Equation (1)) can be used to calculate the signal attenuation in an optimistic world or scenario. In order to overcome the omnidirectional propagation of the radio signal, multiple APs are used to obtain accurate positioning in 2D and 3D environments as shown in Figure 2, where two naïve examples for three APs are shown. Circles have been used to represent the places where a particular RSSI value is detected for a given AP (red circle for $AP_{1}$, blue circle for $AP_{2}$ and green circle for $AP_{3}$). In the first example, the operational fingerprint $fp=\{rssi_{1},rssi_{2},rssi_{3}\}$ represents a case where $rssi_{1}>>rssi_{2}>>rssi_{3}$, whereas the RSSI values are similar ($rssi_{1}\approx rssi_{2}\approx rssi_{3}$) in the second example. In the two examples, a “perfect” unique positioning estimation is provided in the intersection of the three circles.

**Figure 3:**
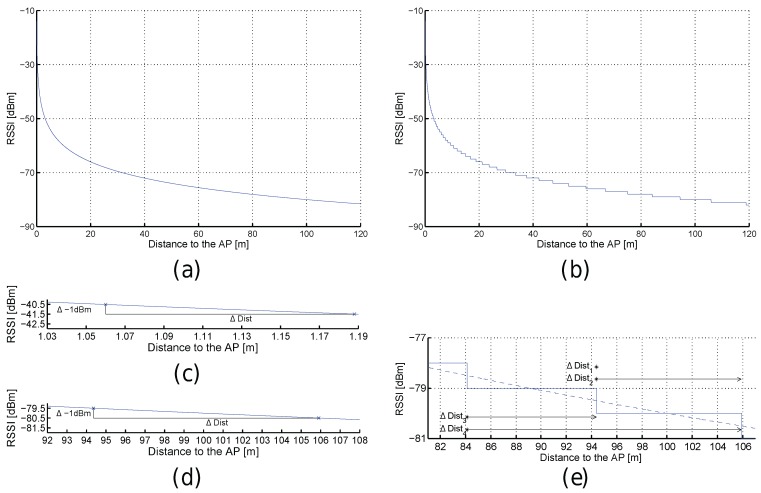
Received signal strength intensities (RSSI) against distance for optimistic (**a**) and quantized (**b**) worlds. (**c**,**d**) Excerpts of (**a**). (**e**) Excerpt of (**b**).

Despite the signal strength being continuous, the devices often report the detected signal level as integer dBm values without decimals. It is worth noting that this quantization only removes the decimal part or rounds to the nearest integer, and does not convert the signal strength to a percentage or quality value. Figure 3 shows some examples of the RSSI according to FSPL. Figure 3a corresponds to the RSSI values from distances comprised between 0 and 120 m from the AP calculated with Equation (1); similarly, Figure 3b shows the values for the quantized world where the RSSI values are rounded to the closest integer value. This quantization step might add uncertainty to the RSSI values.

**Figure 4:**
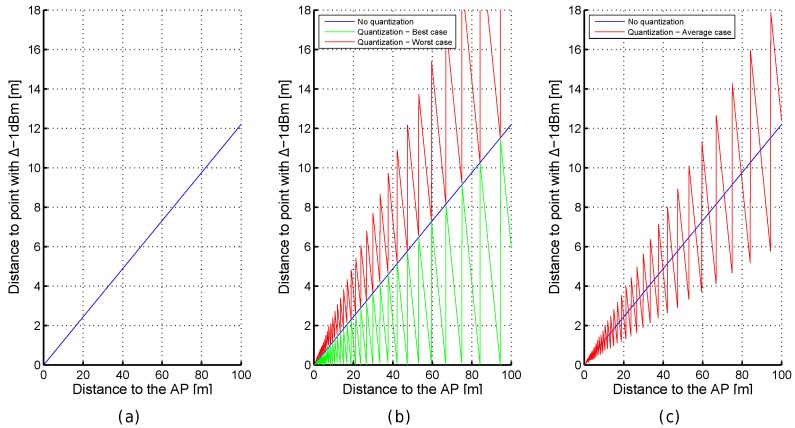
Importance of Δ−1 dBm in the. optimistic (**a**) and quantized (**b**,**c**) worlds

RSSI values are more representative when they are close to the emitter in the optimistic world with neither quantization nor noise; e.g., a decrease of just 1 dBm corresponds to a displacement of ≈13 cm (from −40.5 dBm to −41.5 dBm) but it is ≈11.5 m (from −79.5 dBm to −80.5 dBm) according to Figure 3c,d, respectively. This increment of distance, associated with a decrease of 1 dBm, linearly depends on the distance to the AP as can be seen in Figure 4a.

In the quantized world, the RSSI values are also more representative when they are close to the emitter but rounding them to the closest integer value has a collateral effect: the same RSSI value is seen at a range of distances from the AP (see Figure 3b). Now, the decrease of just 1 dBm has not attached a unique point after rounding the values provided by the FSPL (quantization); e.g., the decrease of 1 dBm from −80 dBm to −81 dBm might be 0 m ($\Delta dist_{1}$ in Figure 3e), ≈11.52 m ($\Delta dist_{2}$ in Figure 3e), ≈10.2 m ($\Delta dist_{3}$ in Figure 3e) or ≈21.7 m ($\Delta dist_{4}$ in Figure 3e). In the best case, the distance (e.g., $\Delta dist_{1}$ and $\Delta dist_{3}$) is lower bounded by 0 m and upper bounded by the distance calculated in the optimistic world. In the worst case, the distance (e.g., $\Delta dist_{2}$ and $\Delta dist_{4}$) is lower bounded by the distance calculated in the optimistic world and upper bounded by approximately twice that distance (see Figure 4b,c). The real RSSI value is shown as a dashed blue line in Figure 3e.

Table 1 is introduced to analyse how quantization might affect fingerprinting. The table shows five representative examples, or cases, at three different base distances to the AP. The RSSI values are compared in the optimistic and quantized worlds.

1. In the first case, a difference of ≈0 dBm in the optimistic world (δRSSI) corresponds to a difference of 0 dBm in the quantized world (δQRSSI) as expected. The real and estimated distance between the two points is almost 0 m as expected.
2. In the second case, a difference of ≈0 dBm in the optimistic world (δRSSI) corresponds to a difference of 1 dBm in the quantized world (δQRSSI). Although the real distance between the two points is 0 m, the estimated distance is higher due to quantization: ≈0.13 m, ≈7.72 m and ≈11.52 m for the three base distances shown in the table.
3. In the third case, a difference of ≈1 dBm in the optimistic world (δRSSI) corresponds to a difference of 0 dBm in the quantized world (δQRSSI). Although the estimated distance between the two points is 0 m due to quantization, the real distance is higher: ≈0.13 m, ≈7.72 m and ≈11.52 m for the three base distances shown in the table.
4. In the fourth case, a difference of ≈1 dBm in the optimistic world (δRSSI) corresponds to a difference of 1 dBm in the quantized world (δQRSSI). The real and estimated distances between the two points match: ≈0.13 m, ≈7.72 m and ≈11.52 m for the three base distances shown in the table.
5. In the fifth case, a difference of ≈1 dBm in the optimistic world (δRSSI) corresponds to a difference of 2 dBm in the quantized world (δQRSSI). The estimated distances between the two points doubles the real distance between them: ≈0.26 m, ≈15.54 m and ≈23.04 m for the three base distances shown in the table.

It is worth noting that the difference between the real and estimated distances depends on the distance with respect to the AP in all cases.

The five cases shown in Table 1 demonstrate that the δQRSSI values have an uncertainty of ±1 dBm, e.g., a difference of RSSI in the real world of 1 dBm might correspond to a difference of RSSI in the quantized world of 0, 1 or 2 dBm. In summary, rounding the RSSI values has introduced some uncertainty which might be considered a source of error. On the one hand, a difference of 0 dBm, which corresponds to a perfect match in the optimistic world, may have attached a large positioning error. On the other hand, a difference of 1 dBm, which might correspond to two separated positions, might correspond to a perfect match. If the RSSI comparison is done close to the antenna, the difference between the real and estimated distances is low (less than 50 cm). However, this difference becomes higher and higher as the distance to the antenna increases (about 7–14 m for points placed at 60 m and 11–23 m for points placed at 95 m) according to the FSPL equation.

**Figure 5:**
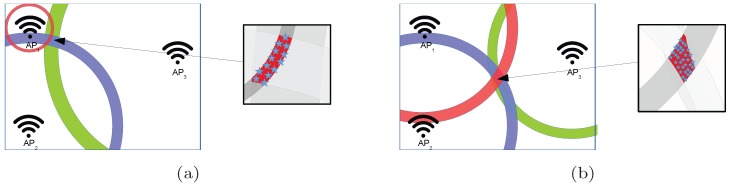
Illustrative examples of the areas where a fingerprint can be placed in the quantized world. (**a**) Fingerprint placed near $AP_{1}$. (**b**) Fingerprint equidistant to all APs.

The uncertainty introduced by the quantization of RSSI values and the omnidirectional signal propagation are partially overcome by the use of RSSI values from multiple APs. Figure 5 shows two naïve examples of fingerprinting for three APs. Rings have been used to represent the area where a particular RSSI value is detected for a given AP since quantization is present (red ring for $AP_{1}$, blue ring for $AP_{2}$ and green ring for $AP_{3}$).

In Figure 5a, the operational fingerprint $fp=\{rssi_{1},rssi_{2},rssi_{3}\}$ represents a case where $rssi_{1}>>rssi_{2}>>rssi_{3}$. It represents the case where the position is near to just one of the APs. In this first example, the ring area for $AP_{1}$ is much smaller than the ring area for $AP_{3}$, since the RSSI value is much higher (stronger). Therefore, the ring area for $AP_{1}$ has the lowest radius (which is related to the FSPL) and width (which is related to the uncertainty due to the distance to the AP), which is in line with the reliability of the estimated distance found in [47]. The small area where this fingerprint can be detected is shown on the right side of the figure. This area is small because of the proximity to one of the APs.

In Figure 5b, the operational fingerprint $fp=\{rssi_{1},rssi_{2},rssi_{3}\}$ represents a case where $rssi_{1}\approx rssi_{2}\approx rssi_{3}$. It represents the case where the distance of all APs to the unknown position is similar. In this second example, the size of the three ring areas is similar. The area where this fingerprint can be detected is higher than for the first example because the aggregated uncertainty considering each individual AP is higher for this particular fingerprint. It is important to note that the same AP distribution has been considered in both examples.

Contrary to the optimistic world where a fingerprint is unique, the same exact fingerprint can be placed within an area (intersection of the three rings) in the quantized world (without noise). This area, considering its size and shape, highly depends on the place where the fingerprint was taken and the AP distribution.

#### 4.1.1. Quantization in the Evaluation Scenario

The previous subsection introduced the uncertainty due to quantization in the determination of the distance to a single AP. In order to analyse the quantization of RSSI values as a source of positioning errors in a realistic indoor environment, some different simulations were carried out. The following default indoor positioning system parameters previously mentioned were applied: Deterministic k-NN algorithm with k=1 as an indoor positioning system; the 50 × 20 m area resembling the PIEP laboratory, the AP distribution with eight APs previously mentioned; and reference fingerprints in a 1 m grid. For the quantized world, only one fingerprint per reference point was generated since fingerprints do not contain any noise (generating the same exact fingerprint multiple times makes no sense in this case). In the radio map, the reference points were placed at the grid intersection points (totalling 1071 − 51 × 21- reference points), whereas each grid cell contained an operational fingerprint in a random position (totalling 1000 − 50 × 20- evaluation points). This procedure was repeated 100 times in order to have a more representative averaged mean error and maximum error. This corresponds to the base simulation setup followed in all the experiments carried out in this section.

Table 2 shows the accuracy of the 1-NN algorithm in the optimistic world (no quantization and no noise) and in the quantized world (no noise). The mean error corresponds to the average of the mean positioning error over the 100 repetitions, the maximum error corresponds to the average of the maximum positioning error over the 100 repetitions, the percentage of cases above the Maximum Expected Error (MEE) corresponds to the number of operational fingerprints whose error was higher than the MEE. Assuming that the 1-NN algorithm returns the best match, the MEE should never be higher than 0.707 m ($\sqrt{0.5^{2}+0.5^{2}}$) for the 1 m grid. This distance corresponds to the highest distance between an operational point and its best match (closest fingerprint in the real-world space).

According to the simulation results shown in Table 2, quantization has a low impact on the mean positioning error, which increases less than 11 cm on average. However, the maximum reported error is almost doubled and the percentage of cases above the maximum expected error (0.707 m for a 1 m grid) increases from 1.367% to 20.222% of cases on average. The increase in the maximum error and the percentage of cases above the maximum expected error shows that quantization has a significant impact on the indoor positioning system since it has introduced large errors which were not present in the optimistic world.

**Figure 6:**
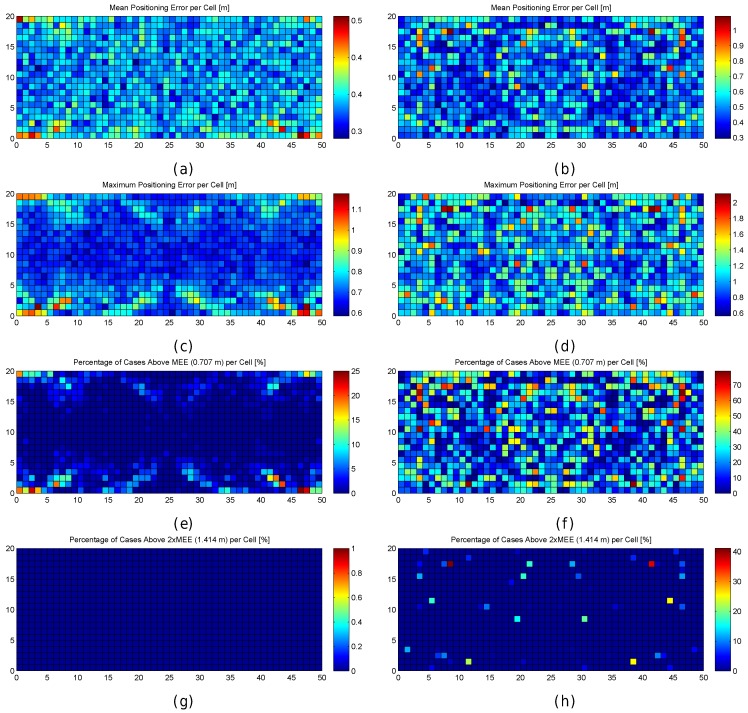
Graphical results for analysing the impact of quantization. (**a**,**b**) mean positioning error per cell in the optimistic and quantized worlds respectively; (**c**,**d**) maximum positioning error per cell in the optimistic and quantized worlds respectively; (**e**,**f**) percentage of cases where the error was higher than MEE per cell in the optimistic and quantized worlds respectively; (**g**,**h**) percentage of cases where the error was higher than twice the MEE per cell in the optimistic and quantized worlds respectively.

In order to better explore the impact of quantization, the results are also graphically shown in Figure 6. In the figure, the mean positioning error, the maximum positioning error and the percentage of cases where the error was above 0.707 m are shown for each cell of the environment in the optimistic and quantized worlds. These results have been calculated after repeating the simulations 100 times, i.e., each cell in the scatter plot shows the metric value that has been calculated using the positioning error over the 100 evaluation points attached to the cell, one for each of the 100 simulations.

For the optimistic world, Figure 6a shows that the mean positioning error is comprised of between 0.35 m and 0.4 m in most of the cells. Only a few cells, located near the corners, provide higher mean positioning errors of around 0.5 m. Similarly, the maximum positioning error per cell (Figure 6c) is comprised of between 0.6 m and 0.75 m in most of the cases. The maximum values (above 1 m) are reached near the corners. Also, the percentage of cases where the error was higher than 0.707 m (Figure 6e) is notably higher in the cells located near the corners with a percentage close to 25%, whereas it is lower than 5% in the rest of the cells. In the optimistic world, the error is never higher than twice the MEE (Figure 6g).

When quantization is introduced, the mean positioning error per cell (Figure 6b) increases with respect to the optimistic world, and the locations of high values are more scattered (Figure 6d). This is also the case for the maximum positioning error per cell and the percentage of cases where the error has been higher than 0.707 m (Figure 6f). Positioning errors higher than 1.414 (twice the maximum expected error) appear and the percentage of these errors is between 25% and 40% (Figure 6h) in some cells.

**Figure 7:**
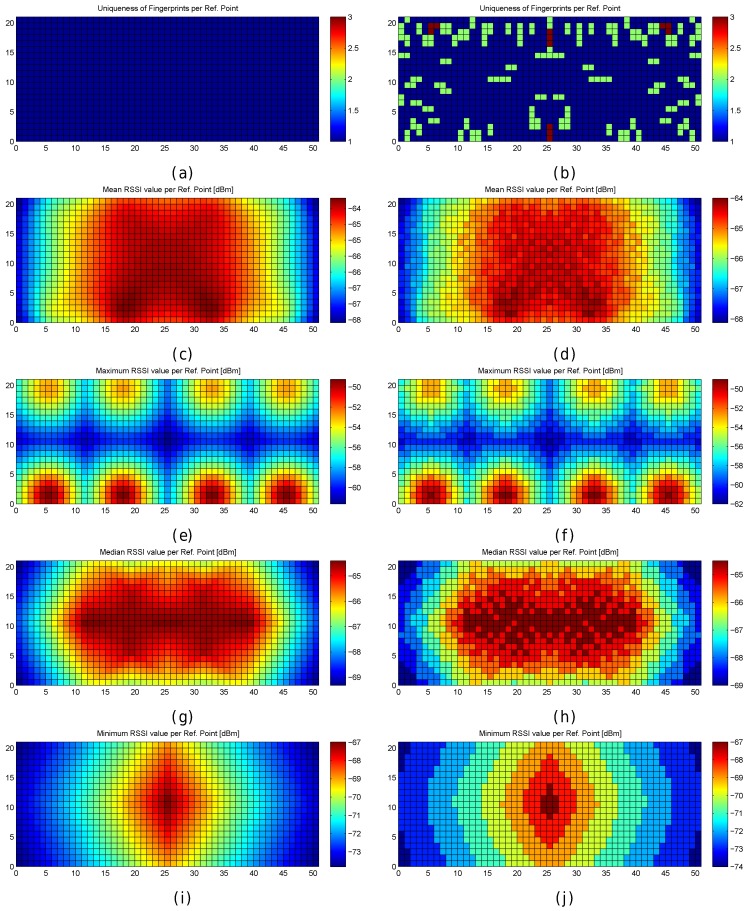
RSSI and fingerprint statistics in the optimistic world (left images) and quantized world (right images). (**a**,**b**) uniqueness of reference fingerprints; (**c**,**d**) mean RSSI value of reference fingerprints; (**e**,**f**) maximum RSSI value of reference fingerprints; (**g**,**h**) median RSSI value of reference fingerprints; (**i**,**j**) minimum RSSI value of reference fingerprints.

Figure 7 graphically shows the following fingerprint statistics for each reference point in the optimistic and quantized worlds: Uniqueness (number of reference fingerprints with exactly the same RSSI values) and mean/maximum/median/minimum RSSI value for each reference fingerprint. According to the figure, the reference fingerprints are unique in the optimistic world, i.e., given any two reference fingerprints, the RSSI vectors never completely match. However, there are some cases where the fingerprints are not unique in the quantized world. In a significant number of cases (green cells in Figure 7b), two fingerprints have exactly the same RSSI values. In four cases (red cells in Figure 7b), three fingerprints have exactly the same RSSI values. The other statistics are similar in the optimistic world and quantized world, with those in the optimistic world being more smoothed.

There is no doubt that quantization has increased the positioning errors. If the statistics of the RSSI values were analysed, it could be observed that most of the large errors are located near the areas where the reference fingerprints were similar and they were not unique.

### 4.2. Noise of RSSI Values

The electro-magnetic signals are affected by many features and elements present in the environment, which introduce reflection, refraction, absorption and diffraction (among others) in the signal. Therefore, the signal strength fluctuates when measured over time at a given position.

The addition of white Gaussian noise is the usual starting point for understanding basic performance relationships in the study of communication systems [48]. Although this assumption is not always true, it significantly simplifies the computations with little performance loss [34]. According to [36], most of RSSI distributions (70% approx.) are often left-skewed, despite the normal distribution being usually used. Although consensus about the best model to fit the RSSI data distributions has not been reached [49], most authors agree that the RSSI histograms resemble a Gaussian distribution in most of the cases. Therefore, in the rest of this paper, we assume that noise in the RSSI values can be modelled by a Gaussian distribution with null mean and standard deviation (σ) between 1 and 5.

**Figure 8:**
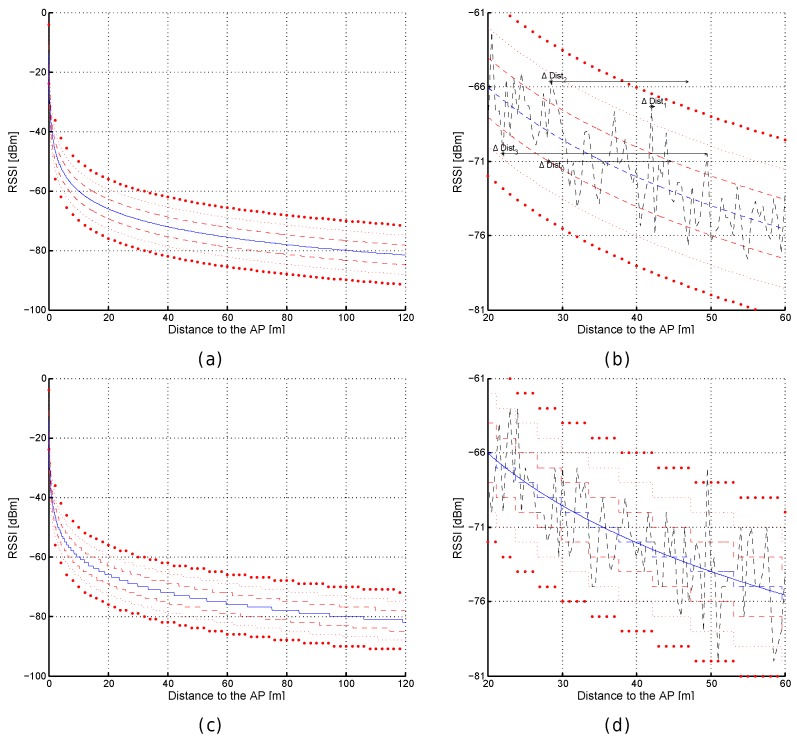
Received signal strength intensities (RSSI) against distance for the realistic noisy world (σ=2) without quantization (**a**) and with quantization (**c**). (**b**) Excerpt of (**a**). (**d**) Excerpt of (**c**).

Figure 8 shows some examples of the RSSI according to the distance to the AP using the path loss with Gaussian noise (σ=2). In particular, Figure 8a shows the signal strength calculated with the path loss model (blue line) plus the bounds corresponding to the 68–95–99 rule (i.e., the 68.27%, 95.45% and 99.73% of noisy RSSI values fall within the first, second and third bounds respectively). Similarly, Figure 8c shows the path loss equation when noise is present (σ=2) and the RSSI values are quantized.

Figure 8b shows an example of the uncertainty introduced by noise. First, $\Delta dist_{0}$ shows the range of distances to the AP where the same RSSI, −71 dBm, can be obtained in 68.27% of cases for the path loss model with σ=2. Although most of the injected noise is close to the zero-mean according to the Gaussian distribution, there might be a few outliers whose error is considerably high (more than three times σ) as occurs in a real scenario (see an outlier example in Figure 8d at 49.5 m from the AP). Second, $\Delta dist_{1}$ shows that two measures taken at close positions might have a large difference in RSSI (≈9 dBm in the example) but the same difference in RSSI might correspond to two separated positions as shown in $\Delta dist_{2}$. Finally, the same RSSI can be detected at two separated positions as shown in $\Delta dist_{3}$. Figure 8d shows an example of the uncertainty in the realistic simulated world, where quantization and noise are both present. In general, Figure 8 shows that the noise present in the radio signal adds more uncertainty in order to estimate a position.

In the rest of this paper, some simulations will be carried out in the optimistic world (no quantization, no noise), in the quantized world (quantization, no noise), and in the realistic word (quantization, noise). The base of all of them is the path loss equation introduced in Equation (1); quantization is just rounding the signal strength to the nearest integer value, and the noise is injected using a normal distribution with zero mean and σ=2 (other values of σ will be occasionally explored). The configuration for the realistic world has been used in many other previous works and we consider that it is a valid configuration to explore the sources of errors, which are inherited from the methods and assumptions in deterministic fingerprinting.

### 4.3. The Grid Size

In order to analyse the grid size as a source of positioning errors, some simulations were carried out. In particular, the following different grid sizes were considered: 10 m, 5 m, 2 m, 1 m, 0.5 m, 0.2 m and 0.1 m. In order to have a more comprehensive study, the analysis considers the optimistic (without quantization and noise), quantized (with quantization and without noise) and realistic (with quantization and noise) worlds.

#### 4.3.1. The Optimistic World

For the optimistic world, the results of applying different grid sizes are shown in Table 3.

According to Table 3, the positioning error depends on the grid size when fingerprinting is applied in the optimistic world without quantization and noise. The lower the grid size, the lower the mean positioning error, which is the common assumption in fingerprinting. However, this table shows an interesting finding, even in the ideal world without noise and quantization, there are a few cases (around 1% and 2% depending on the grid size) where the positioning errors are larger than expected. Another interesting result, is that the average of the maximum error over the 100 simulations is lower than the MEE threshold for the grid size of 10 m. On the other hand, the maximum positioning error almost doubles the MEE for the grid size of 10 cm.

**Figure 9:**
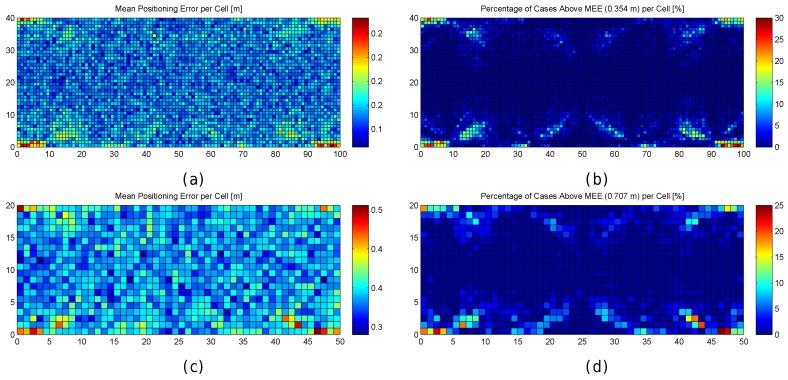
Graphical results for analysing the grid size in the optimistic world. (**a**,**c**) mean positioning error per cell in the optimistic world for a grid size of 0.5 m and 1 m respectively; (**b**,**d**) percentage of cases where the positioning error is above the MEE for a grid size of 0.5 m and 1 m respectively.

In order to better analyse where the large error occurs, Figure 9 graphically shows the mean positioning error per grid cell and the percentage of cases where the positioning error was higher than the maximum expected error; only the 0.5 m and 1 m grid have been considered.

The graphical accuracy shown in Figure 9 clearly depicts a trend in the evaluation scenario, as there are some areas (cells) where the mean positioning error is clearly higher than in the rest; i.e., there are areas where the fingerprints are smoothed and the closest fingerprint in the RSSI space might not correspond to the closest fingerprint in the geometric space. Four of these areas correspond to the scenario corners. Moreover, a geometric pattern is clearly observed as being attached to the antenna position in Figure 9b,d, where the percentage of cases above the maximum expected error is shown.

#### 4.3.2. The Quantized World

Table 4 shows the results for the different grid sizes in the quantized world; the results for the optimistic world were introduced in a similar fashion.

The positioning error also depends on the grid size in the quantized world. However, there is a lower bound in the mean and maximum positioning errors for grid sizes below 1 m. Also, the percentage of cases above the MEE increases for grid sizes below 1 m. Another interesting finding is that the maximum error is very large (more than twice the MEE) for grid sizes lower than 1 m, which indicates that the estimated position is outside the cell delimited by the four surrounding reference points.

**Figure 10:**
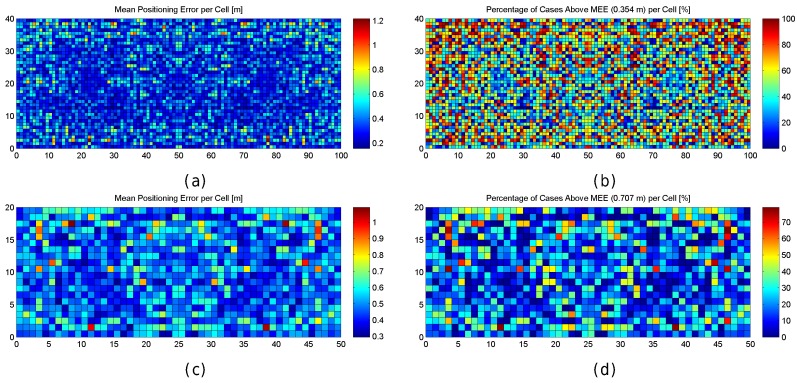
Graphical results for analysing the grid size in the quantized world. (**a**,**c**) mean positioning error per cell in the quantized world for a grid size of 0.5 m and 1 m respectively; (**b**,**d**) percentage of cases above the MEE in the quantized world for a grid size of 0.5 m and 1 m respectively.

Figure 10 graphically shows the mean positioning error per grid cell and the percentage of cases where the positioning error was higher than the maximum expected error for the 0.5 m and 1 m grids.

In the quantized world, there are still some areas where the mean positioning error is clearly higher than in the rest (see Figure 10). When compared to the same figures for the optimistic world (see Figure 9), it can be observed that the number of cases above the maximum expected error has increased. According to the results and the analysis presented in this section, quantization is a phenomenon present in fingerprinting which renders more difficult the task required to differentiate fingerprints collected at nearby positions.

#### 4.3.3. The Realistic Noisy World

Finally, the realistic noisy world is analysed. Table 5 shows the results for the different grid sizes in the quantized world; the results for the optimistic and quantized worlds were introduced in a similar fashion. In this table, five different levels of noise (σ value for the Gaussian distribution) are considered.

In the realistic noisy world (with quantization and noise), the positioning error depends on the grid size and variance of the RSSI values. On the one hand, the lower the grid size, the lower the mean positioning error. On the other hand, the lower the variability of RSSI readings, the lower the positioning error. However, the combination of grid size and signal variability leads to an interesting finding: the grid size has little impact on the mean accuracy of the lower grid sizes as the signal variability becomes high, e.g., the accuracy obtained using a 2 m grid is very similar to the accuracy obtained using a 1 m grid for any σ value, but the number of reference points is four times lower in the former case (2 m grid).

A second interesting finding can be observed in the table: the maximum error and the percentage of cases where the error was higher than the maximum expected error increase as the grid size decreases (except for just one case with grid=10 m and σ=1), which is consistent with the results shown for the quantized world. Also, both increase as the injected noise (σ) increases, i.e., the probability of obtaining an error higher than the MEE depends on the signal variability and it inversely depends on the grid size according to the simulations carried out.

It is important to remark that, contrary to the common rationale, decreasing the grid size does not guarantee a corresponding decrease in the mean positioning error. In fact, the probability of having large positioning errors increases when the grid size is lower than 2 m, which might be due to the high density of fingerprints.

**Figure 11:**
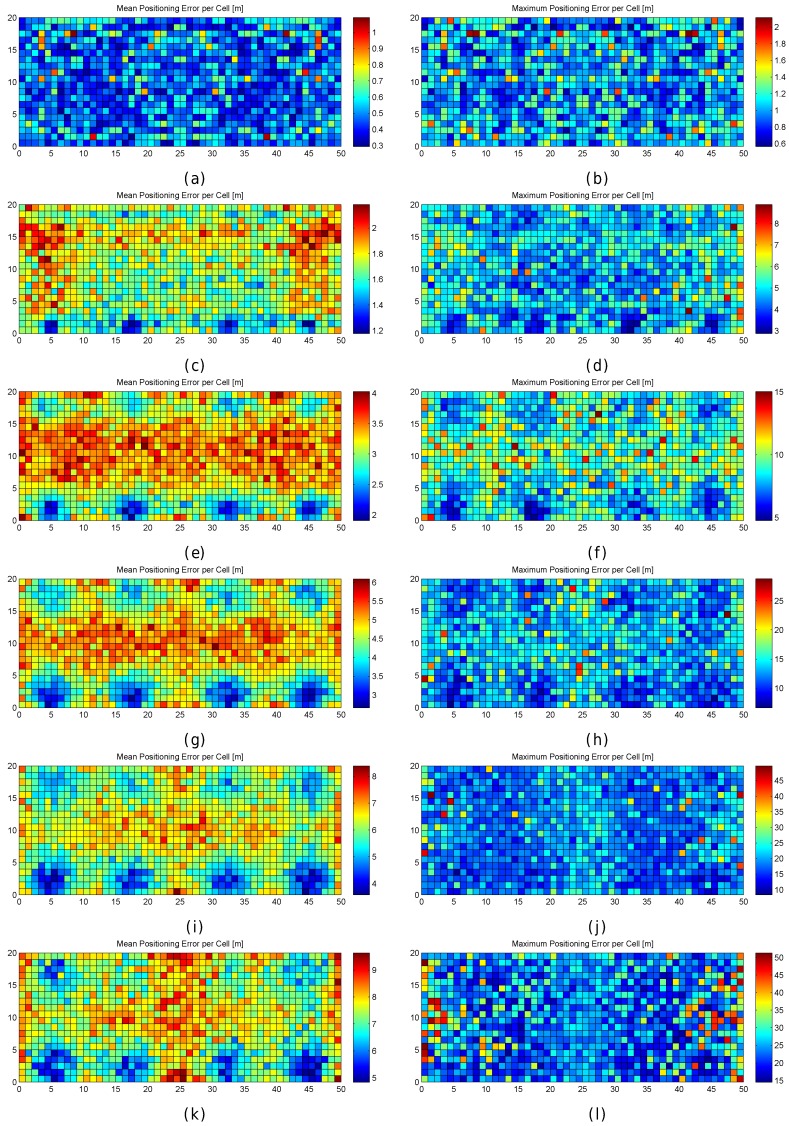
Graphical results (mean and maximum positioning errors over 100 runs) for analysing the grid size in the noisy world. (**a**,**b**) results for σ=0 (no noise); (**c**,**d**) results for σ=1; (**e**,**f**) results for σ=2; (**g**,**h**) results for σ=3; (**i**,**j**) results for σ=4; (**k**,**l**) results for σ=5.

Figure 11 graphically shows the accuracy (as mean and maximum positioning errors) of the 1 m grid configuration and different values of RSSI signal variance (from 0 to 5). This figure clearly shows that the error is not uniform in the grid.

Observing the mean positioning errors depicted in Figure 11, a pattern arises. The areas with low mean positioning error correspond to the location of antennas and their surroundings. The cells providing a large mean positioning error are located in three main parts of the scenario: the horizontal line that divides the scenario into two symmetrical parts, the vertical line that divides the scenario into two symmetrical parts and the cells located in the periphery (especially on the top, left and right sides). The cells close to the horizontal and vertical lines have the lowest maximum RSSI value (see Figure 7e,f), whereas the cells located in the periphery have two interesting features: (1) they are not surrounded by Wi-Fi APs and (2) they are not uniformly surrounded by other reference points because there are not any reference points outside the scenario (i.e., there are less contiguous cells and reference points).

#### 4.3.4. General Discussion about the Grid Size

The previous observed facts about the grid size indicate that the regular division of the environment might not be the best strategy to map an environment since the signal propagation model, the distribution of antennas and other features attached to the signal propagation have not been considered. Even in the optimistic world, it can be seen that the mean and maximum positioning errors are not uniform in all the cells that compose the environment. For instance, the corners in the testing scenario seem more difficult to differentiate.

Some well-established assumptions are not totally true as complexity is added to the operational scenario (quantization and noise), e.g, *“the positioning error decreases as the grid size decreases”*. Small grids are associated with an overall low mean positioning error, but at the expense of having higher maximum errors and more individual cases where the positioning error is higher than the maximum expected error for the corresponding grid size; e.g., in less than 5% of cases, the error was lower than 0.7 m (the maximum expected error for a 1 m grid) for σ equal or higher than 2 (moderate–high variability in RSSI values), but in less than 1% of cases the error was lower than 0.35 m (the MEE for a 0.5 m grid) for σ equal or higher than 3 (moderate–high variability in RSSI values). Also, the accuracy reported with the 2 m grid is similar to the accuracy provided with the 0.5 m grid according to the simulated results.

Furthermore, the simple grid strategy that is usually applied in fingerprinting might be considered a source of positioning error since the RSSI values might be wrongly treated as unitless numbers, as stated in [28]; i.e., the environment is divided according to an arbitrary geometric rule but the physical underpinning of radio signal propagation is ignored when the radio map is generated.

### 4.4. The Density of APs

This section focuses on the number and distribution of APs as a source of positioning errors. The analysis is also done through a comprehensive simulation.

#### 4.4.1. Theoretical Bounds of AP Density

**Figure 12:**
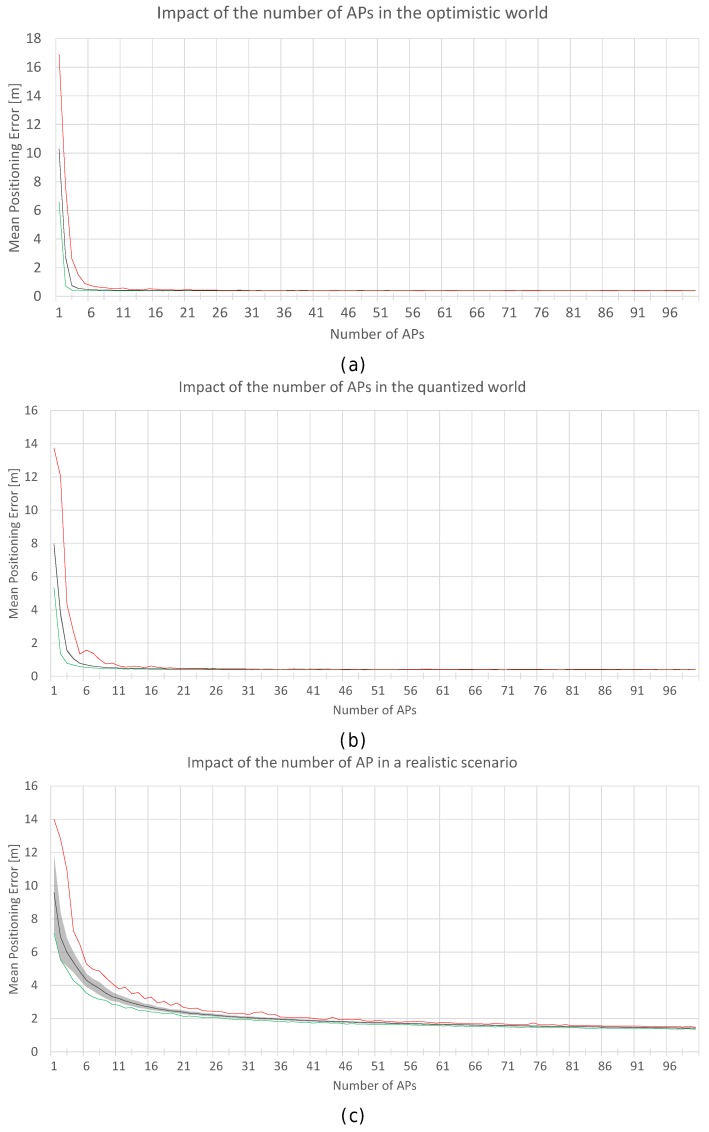
Impact of the number of APs in the optimistic (**a**); quantized (**b**) and realistic noisy (**c**) worlds. Black lines correspond to the average accuracy over the 100 simulations, green lines correspond to min. accuracy, and the red lines correspond to the max. accuracy.

First, the optimal number of APs was established by simulation. The optimistic, quantized and realistic noisy world scenarios were all considered to analyse the effect of AP density on positioning accuracy in depth. Figure 12a shows a plot with the minimum (green), mean (black) and maximum (red) positioning accuracy by using 1 to 100 APs randomly (uniform distribution) distributed in the scenario; the gray area corresponds to the standard deviation of the mean value of the 100 simulations. In order to avoid slanted results, the random AP distribution is different in each of the 100 simulation repetitions. In the figure, the accuracy corresponds to the positioning error in the optimistic world without quantization and noise. Similarly, Figure 12b,c show the same statistics for the quantized and real world (with σ=2).

In Figure 12, it can be observed that there is a lower bound in the positioning accuracy, where adding more APs does not have a huge impact on the accuracy. In the optimistic word, an impressive accuracy is obtained with just three APs if they are placed in the optimal place (minimum error, green line). Adding more APs to the scenario reduces the risk of having a large mean positioning error but there is not a significant improvement in the mean accuracy. In the quantized world, an impressive mean accuracy can also be achieved but the number of APs required is slightly higher (6 APs). In the realistic noisy world (with noise σ=2), where this analysis is crucial, the lower bound is about 25–30 APs and 2 m of mean positioning error, i.e., in order to provide competitive accuracy, at least 0.025 APs per m^2^ are required. If the APs are uniformly distributed to cover this scenario without overlappings, this means that the distance of any operational fingerprint to the closest AP should be ≈3.6 m or lower ($\pi \cdot r^{2}<=\frac{50m\cdot 20m}{25APs}$) considering a 2D scenario. This threshold distance is quite far from the distances to the closest AP in the real scenario, where the distance of 20% of the reference points to the closest AP is more than 8 m. Although the expected mean positioning error with 100 APs (0.1 APs per m^2^) is about 1.5 m, installing such a quantity of APs is not feasible in a real deployment. Theoretically, the positioning error might be reduced by adding hundreds of APs, but the improvement is lower bounded by a mean positioning error of about ≈1.1 m and such deployments are not practical at all.

#### 4.4.2. Impact of AP Distribution

Regarding the AP as a source of error, the AP distribution is also studied in this paper. In particular, we focus on the scenario with eight APs as in the real evaluation scenario where we aim to develop a Wi-Fi-based indoor positioning system. Five alternative distributions (see Table 6) have been considered to show that the AP distribution has a direct impact on the distribution of large errors.

The first configuration is similar to the real AP deployment, but the APs are uniformly distributed to avoid large distances between the APs located in the top and bottom part of the scenario. In the second configuration, the APs are located in the diagonals of the squared scenario. The third configuration is a variant of the previous one. In the fourth configuration, the APs are located at the original position with a random displacement of 1 m radius to avoid symmetries. In the fifth configuration, all the APs are uniformly distributed at the horizontal line that divides the scenario into two parts. The location of the APs can be inferred from the maximum RSSI value shown in Figure 7e,f.

Table 7 shows the results for the six studied AP configurations, the original one of our real scenario and the five proposed configurations. The results show that the first configuration provides the lowest mean error over the 100 repetitions. However, this accuracy is just a number and the error distribution over the environmental area cannot be extrapolated because it is an average value.

**Figure 13:**
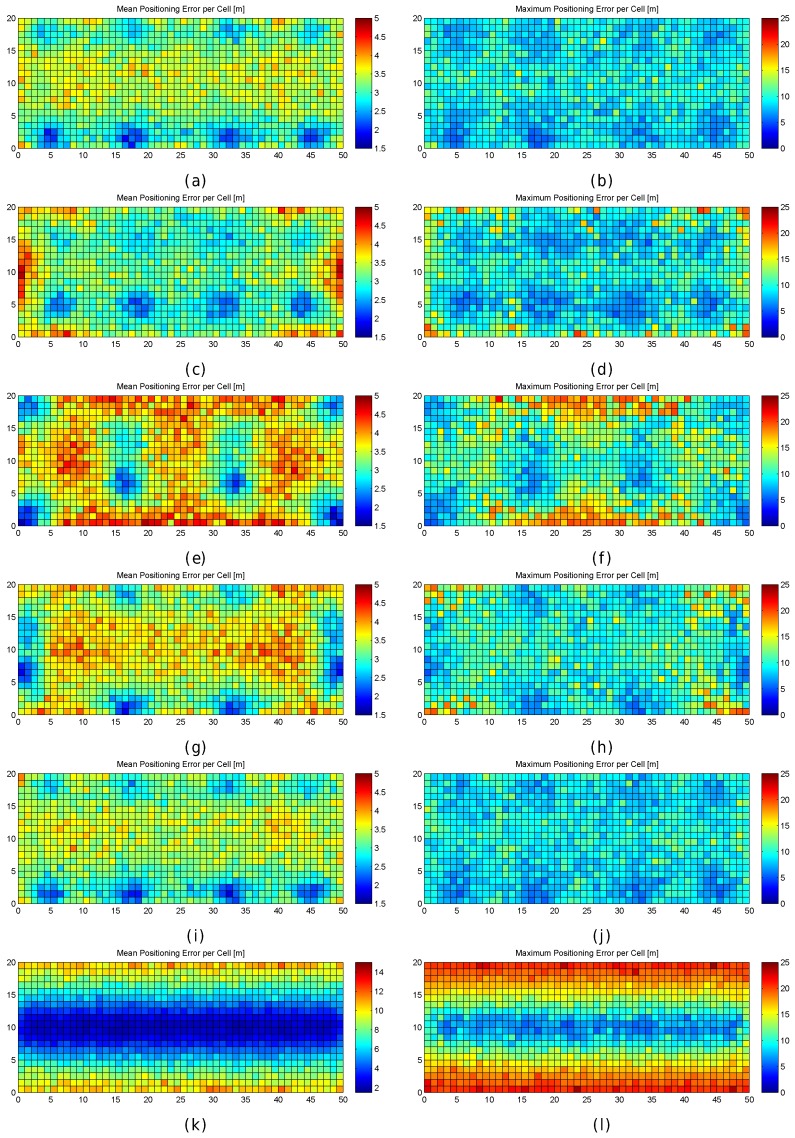
Graphical results (mean and maximum positioning errors over 100 runs) for analysing the AP distribution in the noisy world. (**a**,**b**) original AP distribution; (**c**,**d**) alternative AP distribution 1; (**e**,**f**) alternative AP distribution 2; (**g**,**h**) alternative AP distribution 3; (**i**,**j**) alternative AP distribution 4; (**k**,**l**) alternative AP distribution 5.

**Figure 14:**
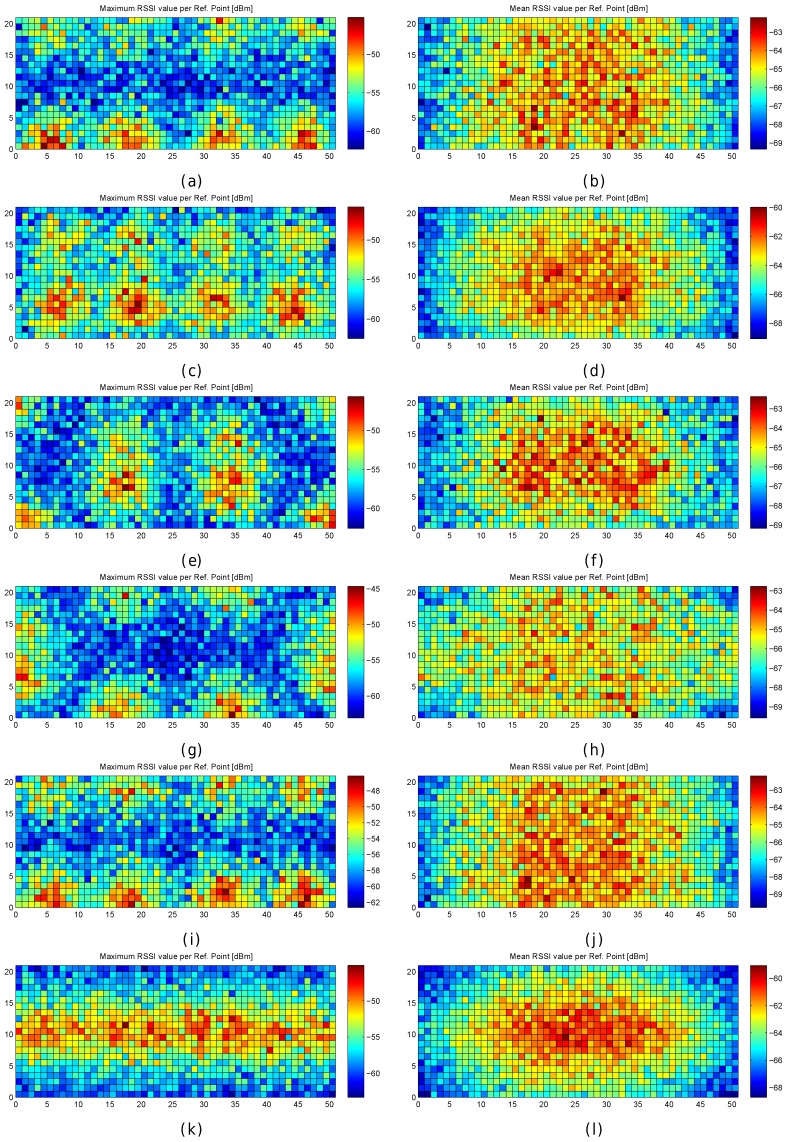
RSSI Statistics (mean and maximum RSSI value) for analysing the AP distribution in the noisy world (σ=2). (**a**,**b**) original AP distribution; (**c**,**d**) alternative AP distribution 1; (**e**,**f**) alternative AP distribution 2; (**g**,**h**) alternative AP distribution 3; (**i**,**j**) alternative AP distribution 4; (**k**,**l**) alternative AP distribution 5.

Figure 13 graphically shows the mean and maximum positioning errors per cell, whereas Figure 14 shows the RSSI statistics (mean and max. RSSI value) for one of the 100 runs. The figures demonstrate how positioning errors are distributed and why large errors occur. Figure 13 clearly shows that the error distribution depends on the locations of the APs (which can be inferred from the maximum RSSI values shown in Figure 14). In the original scenario, with the AP distribution that matches our real environment, the central part of the environment provides the maximum mean positioning error, which is lower than 4 m. In the first alternative, there are many cells located in the left and right sides of the environment which provide a high mean positioning error of about 4.5 m and 5 m, and the maximum error is always lower than 20 m. Similarly, the second and third alternatives show different mean positioning error patterns and also show that they are more prone to having areas in which the maximum positioning error is higher than 20 m (especially in the second alternative). The fourth alternative shows no significant difference with respect to the original AP deployment since the differences in AP location are small. In the last scenario, the effects of symmetry are depicted. Since all the APs are located in a row, it is not possible to determine in which side the fingerprint is located and the errors are quite high in the top and bottom parts of the scenario, where the mean errors are about 15 m, and the maximum errors are about 25 m. To sum up, the positioning error depends on the AP distribution.

When the mean and maximum positioning errors are compared to the statistics of the fingerprints (mean and maximum RSSI value), a pattern again arises. The cells that have a low mean positioning error correspond to the cells which are close to an AP. Moreover, the probability of having a large error is higher in those cells where the mean RSSI value is low.

**Figure 15:**
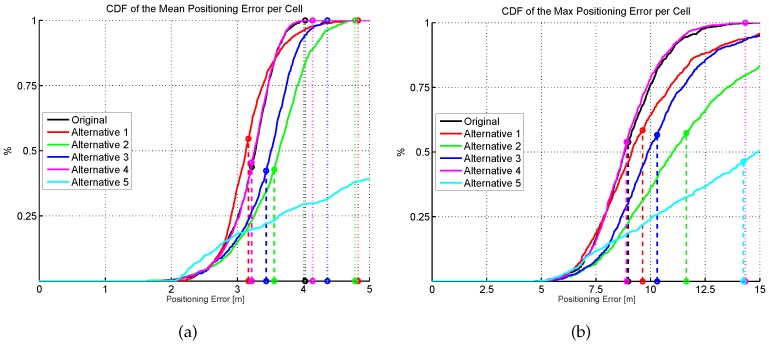
Cumulative distribution of the mean (**a**) and maximum (**b**) positioning error (CDF) for six different AP distributions. Dashed vertical lines indicate the average error, whereas dotted vertical lines indicate the highest error.

The results shown in Figure 13 are summarized in Figure 15, where the CDF of the mean positioning error per cell (100 repetitions) is shown. According to these two plots, the original AP distribution seems to be the best one followed by alternative 4 and alternative 1. Although alternative 1 provides better mean accuracy than the original distribution for more than 75% of cells, it provides worse maximum accuracy than the original distribution.

#### 4.4.3. Presence of Virtual APs

Nowadays, it is common for the same AP to provide different Wi-Fi networks. The reasons for providing multiple networks are diverse: covering different frequencies and channels (2.4 GHz and 5.2 GHz) to provide Internet access to multiple people; providing access to different networks depending on the person’s profile (client, staff, maintenance in a mall); among many others.

Since the AP infrastructure is unknown in some Wi-Fi deployments, the detected networks (RSSI values) are commonly considered as independent networks. In this section, we will analyse the presence of virtual APs as a source of error, but also as a mechanism to improve the accuracy of indoor positioning systems.

For this experiment, it has been considered that each AP can emit up to four independent RF signals (Wi-Fi networks), as this virtual AP configuration has been detected at the University of Minho and Universitat Jaume I. Table 8 shows the mean positioning error for the evaluation scenario with the default AP distribution. An AP distribution with 32 APs is analysed for comparison purposes.

The results of Table 8 show that the default configuration (eight APs, single network) provided a mean positioning error of 3.2 m. By increasing the number of APs to 32, uniformly distributed in the scenario, the error decreases to 1.9 m. Also, the maximum positioning error and the percentage of cases where the error was higher than expected are, both, reduced by using the 32 AP configuration. As previously mentioned, the AP density is higher and, therefore, the IPS performs better.

The table also shows the results of eight APs that emit four different networks each. Here, two options are available: generate a concatenated RSSI vector with 32 values (4 × 8), or generate a vector with eight values by averaging the four RSSI values emitted by each AP (avg × 8). When the four RSSI values emitted by each AP are totally independent, the results are better than with the original eight single-network APs (see rows 3 and 4). Although both alternatives (concatenating and averaging) are good, the averaged solution provides better results. On the one hand, 32 independent APs are detected when the four RSSI values per AP are concatenated, but the accuracy of the 32 uniformly distributed APs is not reached. On the other hand, averaging the values mitigates the noise present in the environment, so the training and operational fingerprint are less affected by noise and the best accuracy in the table is reached, i.e., it is better to have eight APs emitting multiple independent networks than having 32 APs emitting a single network according to the simulations.

When the four APs are partially independent (present a dependency of 50%, e.g., the values from the first and second networks are correlated or identical; and the values from the third and fourth networks are also correlated or identical), the use of virtual APs also improves the accuracy of fingerprinting, but the difference with respect to the original eight single-network APs is lower. If the RSSI values of the four networks are totally correlated (the worst possible scenario for an environment with virtual APs), the accuracy is similar to the original eight single-network APs.

**Figure 16:**
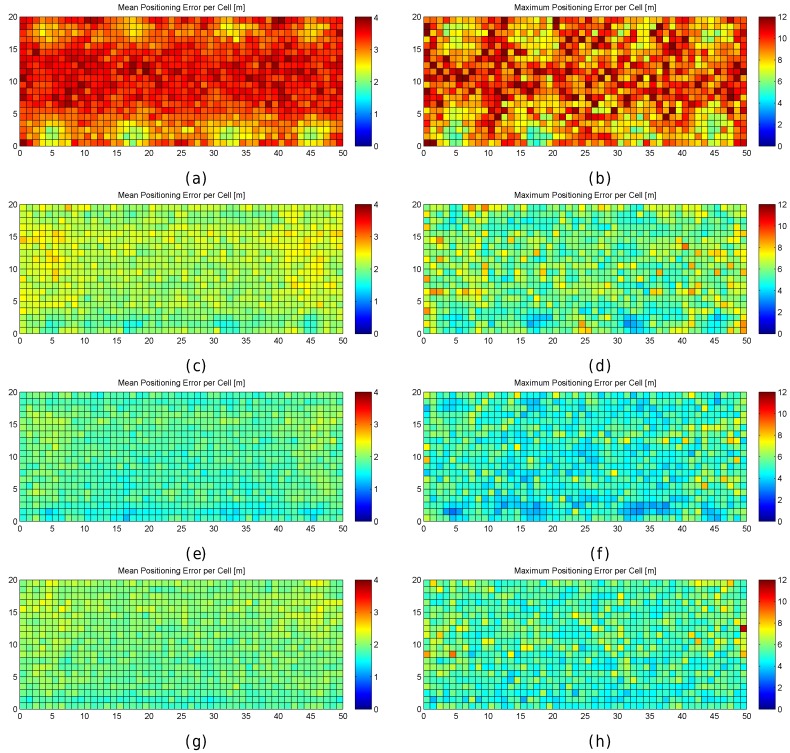
Graphical results (mean and maximum positioning errors over 100 runs) for analysing virtual APs in the noisy world. (**a**,**b**) eight APs, one network per AP; (**c**,**d**) eight APs, four networks per AP (concatenated); (**e**,**f**) eight APs, four networks per AP (average); (**g**,**h**) 32 APs, one network per AP.

**Figure 17:**
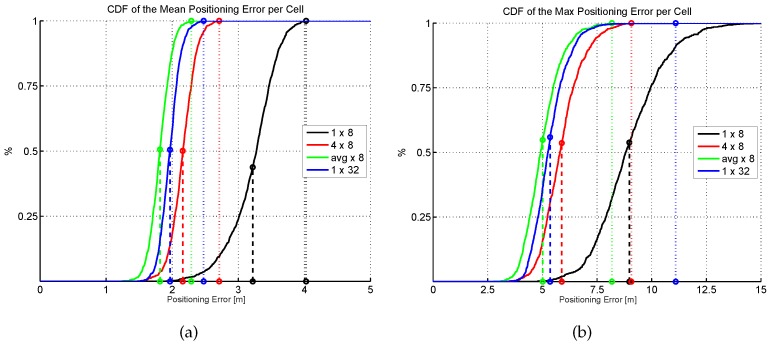
Cumulative distribution of the mean (**a**) and max. (**b**) positioning error (CDF) for comparison of virtual APs. Dashed and dotted vertical lines stand for average and highest error, respectively.

Figure 16 shows the mean and maximum positioning error per cell to compare the results provided without virtual APs (8 and 32 APs) and with virtual APs (concatenating them and averaging them). Figure 17 shows the CDFs of these four configurations in the best possible scenario for the virtual APs (the four RSSI values are not correlated).

It seems that having virtual APs benefits fingerprinting since the presence of outlier RSSI measurements might be reduced. However, in most fingerprinting solutions, virtual APs are not exploited and they are considered as independent networks. Not exploiting this information might be considered a source of error since the RSSI readings remain noisy and the density of APs is artificially increased.

#### 4.4.4. General Discussion about the AP Density

First, the AP density might be considered a source of large errors in Wi-Fi fingerprinting. If the number of detected APs is low, the probability of having large errors is higher since there is not enough information to distinguish fingerprints in a scenario.

The distribution of APs is also important. In those areas where the distance to the closest AP is higher than a threshold, the probability of having a large error increases. In our simulated scenario, the threshold value was ≈3.568 and the largest errors were located in those areas where the distance to the closest APs was larger than this threshold. Hopefully, this source of error can be detected at the operational fingerprint by its maximum RSSI value.

Regarding the presence of virtual APs, they have to be carefully managed. If the virtual APs are considered as independent networks, the AP density is artificially increased and the positioning error is slightly larger than expected, i.e., 32 uniformly distributed APs provide better accuracy than eight uniformly distributed APs emitting four networks. However, the best results are obtained if the knowledge about virtual APs is exploited, i.e., eight averaged uniformly distributed APs provide better accuracy than 32 single-net uniformly distributed APs.

### 4.5. Number of Fingerprints

In order to analyse the number of fingerprints, or FP density, as sources of positioning errors, some different simulations were carried out in a similar fashion as introduced in the previous studies.

#### 4.5.1. Theoretical Bounds of Reference FP Density

First, the optimal number of fingerprints was established by simulation. For this fingerprint feature, only the real world scenario was considered to analyse the effect of reference FP density on positioning accuracy in depth. In the optimistic and quantized world, it makes no sense to collect multiple fingerprints on the same place since the same exact fingerprint is replicated.

**Figure 18:**
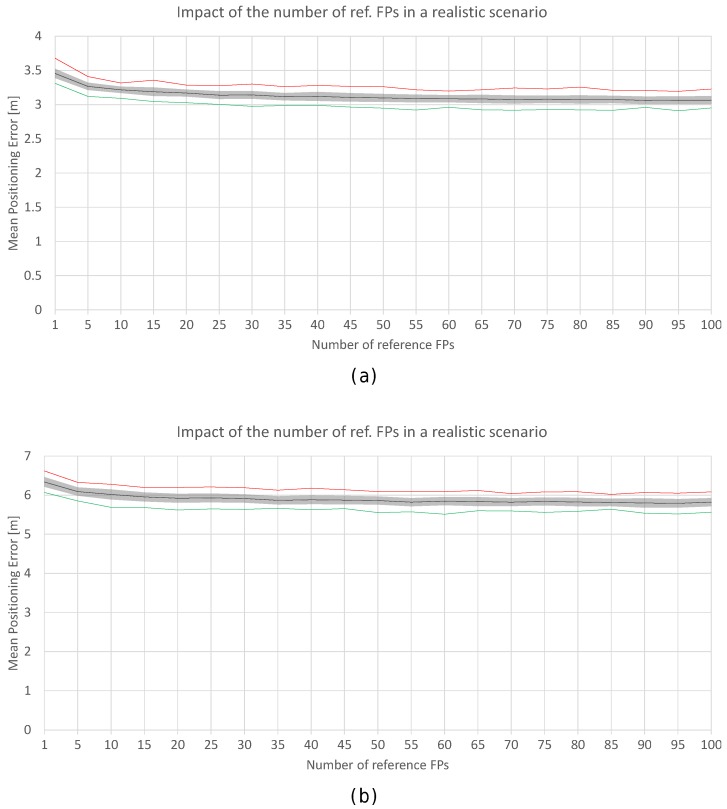
Impact of the number of reference fingerprints in the realistic world with noise σ=2 (**a**) and σ=4 (**b**). Black lines correspond to the average accuracy over the 100 simulations, green lines correspond to min. accuracy, and the red lines correspond to the max. accuracy.

Figure 18 shows a plot with the minimum (green), mean (black) and maximum (red) positioning accuracy by using 1 to 100 FPs per reference point (gray areas represent the standard deviation of the mean positioning accuracy). The minimum, mean and maximum values are calculated based on the performance over the 100 runs. In the figure, the accuracy corresponds to the positioning error in the realistic noisy world for σ=2 (Figure 18a) and σ=4 (Figure 18b).

In Figure 18a, it can be observed that there is a lower bound in the accuracy when σ=2. The mean positioning accuracy is about 3.5 m with just one fingerprint per reference point (3.7 m in the worst run) and 3.1 m with 100 fingerprints per reference point (2.95 m in the best run). With 10 fingerprints per reference point, a common value, the accuracy is about 3.20 m (oscillating between 3.1 and 3.3 in the best and worst runs respectively). It seems that the FP density per reference point has a low impact on the accuracy since the variance of the RSSI signal is 2: the mean positioning error oscillates between ≈3 m and ≈3.7 m. However, it is worth mentioning that the operational fingerprint is just one vector of the RSSI values which might contain outlier values due to the noise. For σ=4, the trends are similar but the mean positioning error is higher (around 6 m).

**Figure 19:**
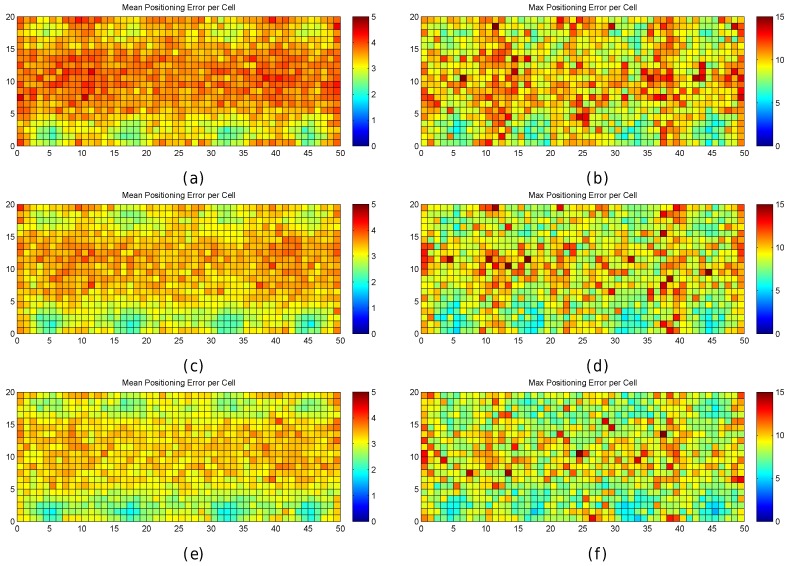
Graphical results (mean and maximum positioning errors over 100 runs) for analysing the AP distribution in the noisy world. (**a**,**b**) one fingerprint per reference point; (**c**,**d**) 10 fingerprints per reference point; (**e**,**f**) 100 fingerprints per reference point.

To better analyse the distribution of the errors, Figure 19 shows the mean and maximum positioning errors per cell for 1, 10 and 100 fingerprints per reference point. For simplification, only σ=2 is considered.

Figure 19 clearly shows that the mean and maximum positioning errors decrease as the number of fingerprints per reference point increases. Although the position of large errors does not vary, the probability of obtaining such errors decreases. However, the difference in the accuracy between 10 and 100 fingerprints per reference point is marginal. It seems that the density of fingerprints per reference point is also a source of large errors. This is a parameter that should be carefully set since a high density of fingerprints per reference point might not drastically reduce large positioning errors and the computational cost might be prohibitive.

#### 4.5.2. Averaging the Reference Dataset

According to the results shown in the previous subsection, it seems that adding more reference fingerprints does not have the desired impact on the IPS accuracy, and the computational cost of k-NN-based indoor positioning systems might significantly increase. However, one can reduce the number of fingerprints by averaging them, i.e., forming a new fingerprint by averaging the RSSI values provided by *n* fingerprints at the same place.

**Figure 20:**
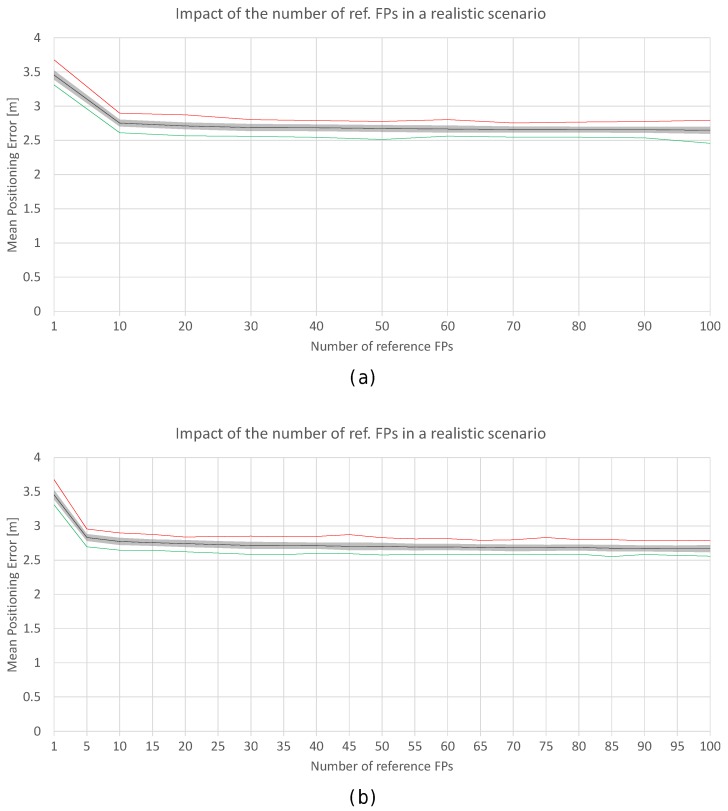
Impact of the number of reference fingerprints in the realistic world with averaging in blocks of 5 (**a**) and averaging in blocks of 10 (**b**). Black lines correspond to the average accuracy over the 100 simulations; green and red lines correspond to min. and max. accuracy, respectively.

Figure 20a shows the positioning accuracy by using 1 to 100 FPs, where the values have been averaged into blocks of five non-overlapping fingerprints. In the plot, ‘100 (individual) fingerprints’ corresponds to ‘20 averaged fingerprints’. Similarly, Figure 20b also shows the positioning accuracy by using 1 to 100 FPs, but the values have been averaged in blocks of 10 non-overlapping fingerprints (‘100 (individual) fingerprints’ corresponds to ‘10 averaged fingerprints’). This procedure might reduce the noise and size of the radio map.

In Figure 20a, it can be observed that the lower bound shown in Figure 18 persists. Averaging the reference fingerprints reduces the mean positioning error to about 2.6 m, which is significantly lower than the accuracy shown in Figure 18. With just one averaged fingerprint per reference point (averaging five independent individual fingerprints), the mean positioning accuracy is about 2.8 m (2.9 m and 2.7 m in the worst and best case respectively) and 2.7 m with 20 averaged fingerprints per reference point (2.8 m and 2.6 m in the worst and best case respectively). For the case of 10 individual independent fingerprints per reference point, the error is reduced by ≈15%, since the accuracy was about 3.20 m before averaging and it is 2.77 m when fingerprint averaging is applied.

In Figure 20b, it can be observed that the accuracy is not highly improved with respect to the results shown in Figure 20a. The reported accuracy and the lower bounds are similar. With just one averaged fingerprint per reference point (averaging 10 independent single fingerprints), the mean positioning accuracy is about 2.75 m (2.9 m and 2.6 m in the worst and best case respectively) and 2.65 m with 10 averaged fingerprints per reference point (2.8 m and 2.45 m in the worst and best case respectively). For 10 individual independent fingerprints per reference point, the accuracy was about 3.20 m before averaging and it is 2.75 m when the fingerprint average is applied.

Increasing the number of reference fingerprints, averaged or not, has a lower bound in the mean and maximum positioning error as can be seen in Figure 18 and Figure 20. Adding more fingerprints has entailed an increase in the computational cost at the training (collection of fingerprints) and operational (estimate position) stages. However, the increase in accuracy might not be as expected. Although the noise in the reference fingerprints is mitigated by averaging, there is a source of positioning error which is not covered: the noise present in the operational fingerprint.

**Figure 21:**
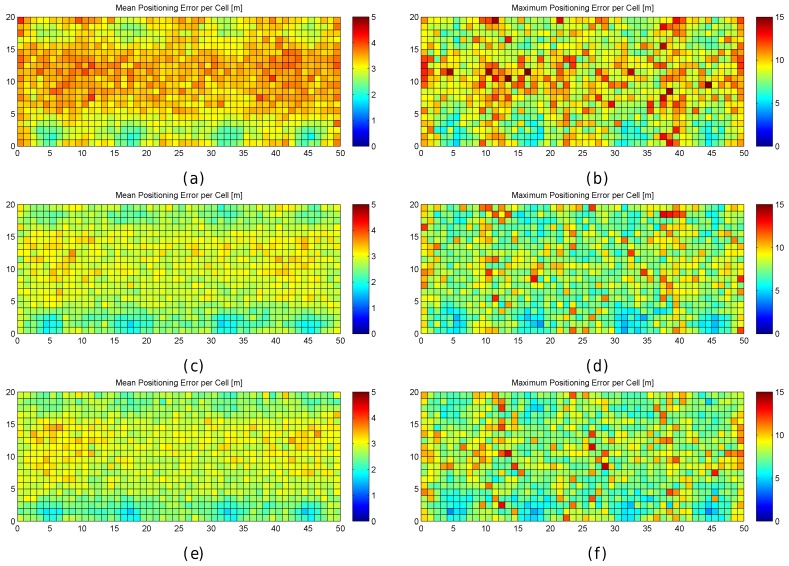
Graphical results (mean and maximum positioning errors over 100 runs) for analysing the impact of averaging reference fingerprints in the realistic noisy world. (**a**,**b**) 10 fingerprints per reference point without averaging; (**c**,**d**) 10 fingerprints per reference point with averaging in blocks of 5; (**e**,**f**) 10 fingerprints per reference point with averaging in blocks of 10.

To better analyse the distribution of the errors, Figure 21 shows the mean and maximum positioning errors by using 10 independent single reference fingerprints, two averaged fingerprints (average of five independent single fingerprints) and one averaged fingerprint (average of 10 independent single fingerprints). For simplification, only σ=2 is considered.

Figure 21 shows that averaging reduces the mean and maximum positioning error per cell. Although the positions of large errors do not vary, the probability of obtaining such errors decreases. Also, the difference between averaging in blocks of 5 and 10 fingerprints is low when 10 independent single fingerprints were collected at the training stage. It is worth noting that the computational costs at the operational stage are approximately 10 times lower in the last case (averaging in blocks of 10 fingerprints) than in the first case (no fingerprint average).

#### 4.5.3. Averaging the Operational Fingerprints

This subsection introduces the last study on the number of fingerprints: averaging multiple fingerprints collected at the operational stage. Averaging operational fingerprints is not common since it requires the node to remain static in a position or to have multiple synchronized interfaces to collect data [46]. However, we consider that its study is also relevant to determine the sources of positioning errors.

Table 9 shows the results of (1) traditional deterministic fingerprinting without averaging (rows 1 and 2); (2) fingerprinting where averaging has only been applied to operational fingerprints (rows 3 and 4); (3) fingerprinting where averaging has only been applied to the reference dataset (rows 5 and 7); and (4) fingerprinting where averaging has been applied to the reference dataset and also in the operational stage (rows 6, 8, 9 and 10).

Table 9 shows that the accuracy is reduced from 3.4 m to 3.2 m by using 10 fingerprints per reference point instead of just 1 (lines 1 and 2); this reductions is about 24 cm. If averaging is applied to the training set only, the accuracy is reduced to ≈2.76 (lines 5 and 7). If averaging is applied to the operational fingerprints only, the accuracy is reduced to ≈2.4 m (lines 3 and 4). This finding in the simulation is relevant: it seems that efforts should concentrate on removing the noise present in the operational fingerprints rather than in the training ones according to the results shown. This makes sense since averaging the training or reference fingerprints reduces the number of candidates for the 1-NN algorithm, i.e., the probability of getting the appropriate closest match is high, if the number of candidates (reference fingerprints) is high.

The best results are obtained when averaging is applied to the training and operational fingerprints (lines 6 and 8 in Table 9), with an impressive mean positioning error of 1.687 m (averaging in blocks of 5) and 1.336 (averaging in blocks of 10). The higher the number of averaged fingerprints, the lower the error. However, it seems that there is a lower bound in accuracy of about 1.5 m (when averaging in blocks of 5) and 1.1 m (when averaging in blocks of 10) as shown in the two last cases (lines 9 and 10) for a radio map composed of 100 individual reference fingerprints that have been averaged in blocks of 5 and 10 non-overlapping fingerprints.

**Figure 22:**
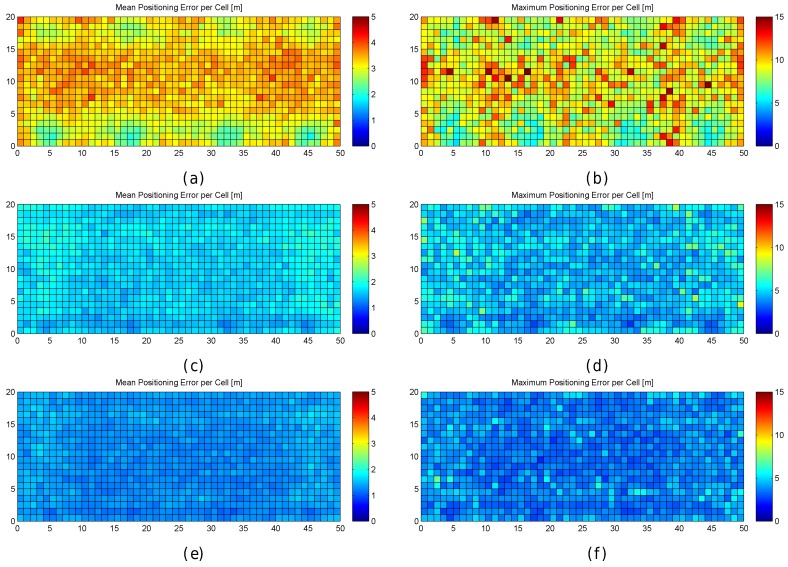
Graphical results (mean and maximum positioning errors over 100 runs) for analysing the impact of averaging training and operational fingerprints in the realistic noisy world. (**a**,**b**) 10 fingerprints per reference point without averaging; (**c**,**d**) 10 fingerprints per reference point with averaging in blocks of 5; (**e**,**f**) 10 fingerprints per reference point with averaging in blocks of 10.

Finally, averaging the reference and operational fingerprints not only reduces the mean positioning error, it also reduces large errors and the percentage of cases where the error was higher than the MEE as shown in Table 9 and Figure 22.

#### 4.5.4. General Discussion about the Number of Fingerprints

First, it seems that having a reasonable number of fingerprints per reference point (e.g., ≈10 in a realistic scenario with σ=2) is enough. Adding hundreds and hundreds of fingerprints per reference point makes no sense in a short period of time: the accuracy is not highly improved since the operational fingerprint is just 1; and the computational costs (at training and operational stages) increase. The accuracy has a lower bound which corresponds to the noise of the operational fingerprint, i.e., if it is an outlier fingerprint, the probability of correctly estimating its position is too low.

Averaging the reference fingerprints improves the accuracy of the indoor positioning systems according to the simulation results. It seems that the noise present in the radio map is significantly reduced and the reference fingerprints are more robust. However, a lower bound in the accuracy persists. It seems that averaging hundreds and hundreds of fingerprints per reference point also makes no sense.

The final step regarding fingerprint density is to increase the number of operational fingerprints and average them. This is not common in indoor navigation solutions since the minimum time between two fingerprints is usually between 1 and 4 seconds according to prior experiments. However, it is feasible if the mobile node remains static in one place or has multiple synchronized interfaces to collect simultaneous fingerprints. Averaging a few operational fingerprints reduces the noise at the operational stage and, therefore, the positioning results are much better since the probability of having an outlier operational fingerprint is much lower.

### 4.6. Estimation Algorithm

This subsection deals with the study of different estimation algorithms used in fingerprinting.

#### 4.6.1. 1NN vs. kNN

**Figure 23:**
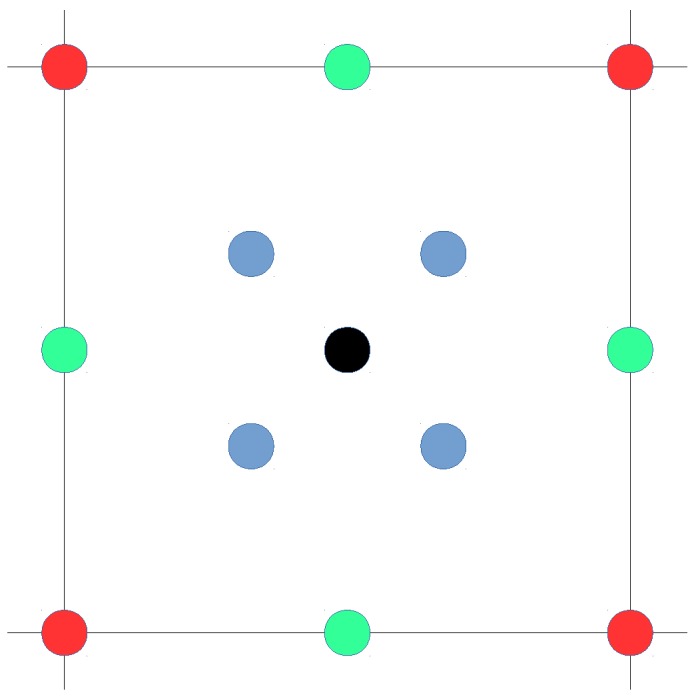
Expected solutions of kNN in the optimistic world for k=1 (red); k=2 (green and black); k=3 (blue); k=4 (black).

The deterministic kNN algorithm is the base algorithm used by many indoor positioning systems. As has already been mentioned, the position is estimated using the k most similar fingerprints from the reference dataset (or radio map). In the optimistic world, where there is only one fingerprint per reference point, the possible estimations for any fingerprint inside a cell are shown in Figure 23. From the distribution of candidates, the best accuracy is expected to be obtained for *k* = 3 since the greatest distance from any fingerprint to the closest candidate is the lowest for the four possible values of *k*. Also, higher values of *k* should not be suitable since a fingerprint is surrounded by just four fingerprints. A similar behaviour is expected in the quantized world.

**Figure 24:**
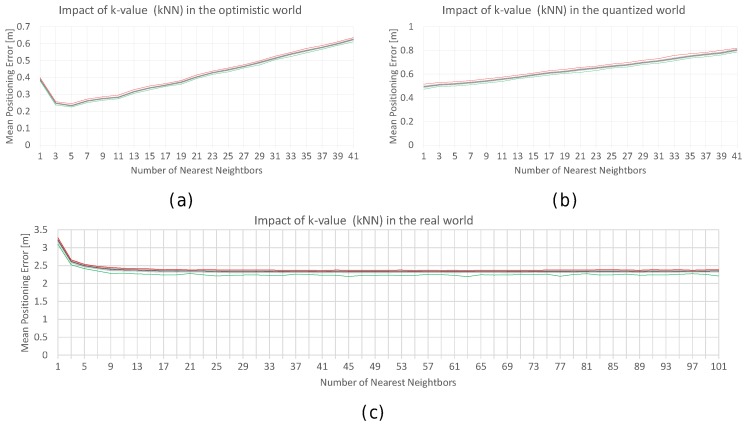
Impact of the number of reference fingerprints in the optimistic (**a**); quantized (**b**) and realistic with σ=2 (**c**) worlds. Black, green and red lines correspond to mean, min. and max. accuracy, respectively.

Several values of *k* ({1,3,5,7,…,39,41}) have been tested in the optimistic and quantized worlds, and the results are shown in Figure 24. The plot for the optimistic world shows that the optimal value of *k* is about 3 to 5 nearest neighbours, whereas 1 seems to be the best *k* value in the quantized world.

For the realistic world, selecting the best value for *k* is slightly different. First, any operational fingerprint is surrounded by 40 fingerprints (10 fingerprints per reference point) instead of just four fingerprints as in the optimistic and quantized worlds. Second, outlier reference and operational fingerprints might appear in the scenario due to the nature of the injected noise. For the realistic world, and assuming a noise with σ=2, more *k* values have been tested ({1,3,5,7,…,99,101}), and the results are shown in Figure 24. Although the plot shows that the optimal values of *k* are in the range of 7 to 41, k=7 is good enough and the difference with the other *k* values is not so high.

The previous figures have shown the mean accuracy of the k-NN algorithm for different values of *k*. Table 10 expands on these figures and shows more information about the maximum positioning error and the percentage of cases above the MEE for the realistic world. According to the table, the lowest mean positioning error is obtained with large *k*-values. In fact, the best value is provided with k=41, where the mean positioning error is 2.328 m. Also, the lowest maximum error is provided by large *k*-values. However, the use of low *k*-values (e.g., k=41) is common in the literature.

#### 4.6.2. Distance Metrics

k-NN is a well-known algorithm that has been used as the basis in many indoor positioning systems. One crucial parameter of k-NN is the distance or similarity measure, which is used to compare two fingerprints.

In order to analyse the effect of the distance metrics on fingerprinting, we compare the accuracy of 41 alternatives [50] in the optimistic and quantized world first. Table 11 shows the mean accuracy, the mean maximum error, the percentage of cases above the MEE for the 1 m grid, and the Pearson correlation coefficient between the feature space (FS) distance and the positioning error.

For the optimistic world, it can be observed that only a few distance metrics are not suitable for fingerprinting, since they report a huge average error. Moreover, the *city block* (or *Manhattan*) distance seem to be better alternatives than the *Euclidean distance*, which is the default distance metric in many fingerprinting approaches. Overall, it seems that alternative distances might be better than the *Manhattan* distance in the optimistic world because they provide lower maximum error, lower percentage of cases where the positioning error was lower than the expected threshold and higher correlation (feature space distance vs. geometric distance). The most significant finding is that none of the metrics analysed is able to assign the closest candidate in the geometric space as the best match for all the operational fingerprints. None of the studied distance metrics, some of them widely used in fingerprinting, is able to fit the meaning of the RSSI values.

For the quantized world, the best approach is the *Euclidean distance* according to the mean positioning error, but the *chebyshev distance* provides lower maximum error. However, the correlation values (distance FS vs. geometric distance) decreased after quantization. Also, the percentage of cases where the error is above the MEE is slightly higher than for the optimistic world. Although the number of unique reference fingerprints in the quantized scenario is ≈80%, the percentage of cases where the error is higher than expected is about 55%.

**Figure 25:**
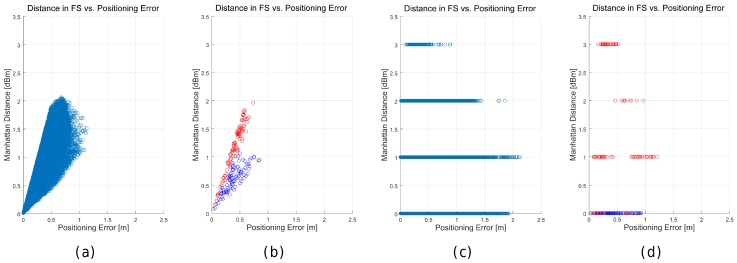
Relation between the distance in the feature space (Manhattan)—y axis—of the best match and the positioning error—x axis—for the happy world (**a**,**b**) and the quantized world (**c**,**d**). (**a**,**c**) show all the tuples generated in the 100 simulations in all the scenarios; whereas (**b**) and (**d**) show the tuples generated in the 100 simulations in two representative cells.

Figure 25 shows the relation between the distance in the feature space (Manhattan) of the closest match and the positioning error for the optimistic and quantized worlds through four scatter plots. Two examples are shown for each world: considering all the results provided in the 100 simulations, and considering two representative cells (red and blue circles in the plots) in the 100 simulations.

In the optimistic world, it can be seen that, as expected, there exists a relation between distance in the feature space (Manhattan distance) and geometric distance (error). Although this relation is not perfect, the distance in the feature space might be considered an estimator of the committed error: a low Manhattan’s distance corresponds to low errors whereas high values correspond to high errors.

However, when quantization is present in the RSSI values, this assumption is no longer true, as can be observed in Figure 25c,d, where low positioning errors (about 0 m) have large distance in the FS, and low distances in the FS (about 3 dBm) provide accurate positioning. Similar behaviours have been provided by other distance metrics such as *Euclidean distance* and *Mahalanobis distance*, which are widely used in fingerprinting.

**Figure 26:**
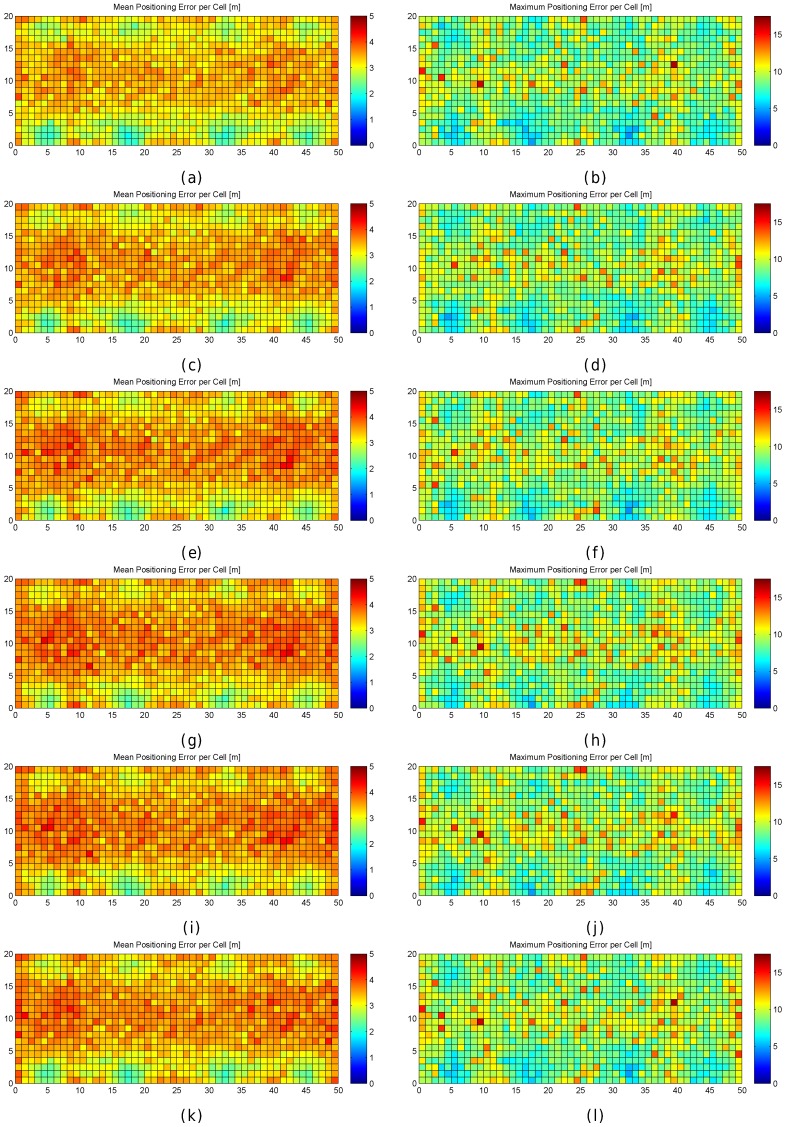
Graphical results (mean and maximum positioning errors over 100 runs) for analysing the distance/similarity metric in the noisy world for 1-NN. (**a**,**b**) Manhattan distance; (**c**,**d**) Euclidean distance; (**e**,**f**) Mahalanobis distance; (**g**,**h**) Matusita distance; (**i**,**j**) Neyman distance; (**k**,**l**) Sorensen distance.

**Figure 27:**
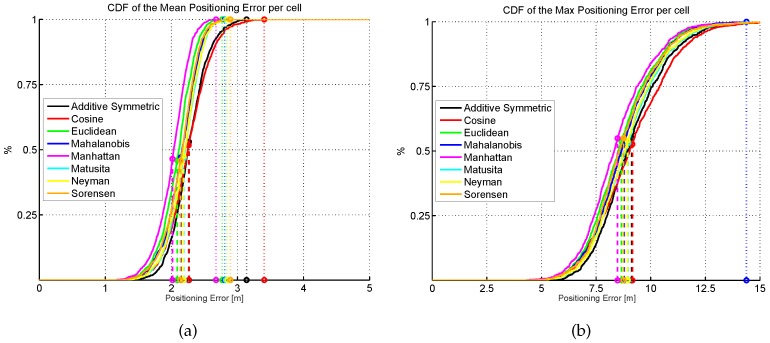
Cumulative distribution of the mean (**a**) and maximum (**b**) positioning error (CDF) for eight different distance/similarity metrics. Dashed vertical lines indicate the average error, whereas dotted vertical lines indicate the highest error.

For the realistic world, the study is limited to only those distance metrics used in the literature, whose results are shown in Table 12. Figure 26 and Figure 27 show more details for a selection of them.

The results provided by the eleven selected metrics are similar, with a mean positioning error between ≈3.2 and ≈3.6 m. However, the most important finding is that the Pearson’s correlation factor is approximately 0 in all of them; i.e., there is no correlation between the distance in the feature space and the geometric distance (positioning error) for any of the selected distance/similarity metrics.

**Figure 28:**
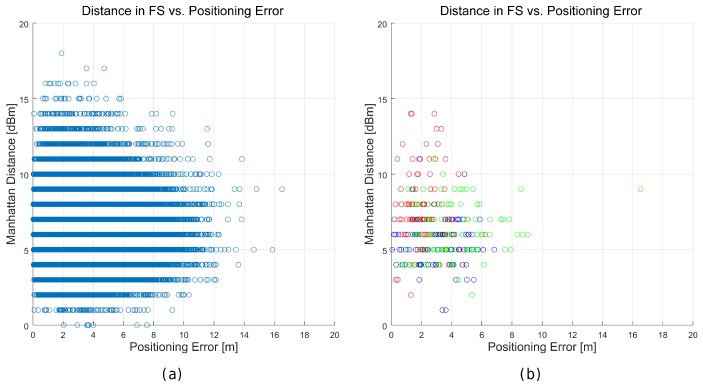
Relation between the distance in the feature space (Manhattan) of the best match and the positioning error for the realistic world. (**a**) all the tuples generated in the 100 simulations; (**b**) tuples generated in the 100 simulations in three representative cells.

This last finding shows that it might be complex to estimate the positioning error from the distance in the feature space. Moreover, it shows that the weights calculated in weighted k-NN based algorithms might be not appropriated, since it seems that there is not a perfect relation between the geometric distance and feature space (RSSI) distance for two compared fingerprints. Figure 28 shows the relation between the distance in the feature space (Manhattan) of the closest match and the positioning error in a realistic world scenario (σ=2) in a similar way as done for the optimistic and quantized worlds (all cases and three representative cells). According to the figure, the tuples are scattered and there is no correlation between the distance in the feature space and the geometric distance between an operational fingerprint and the closest reference fingerprint.

**Figure 29:**
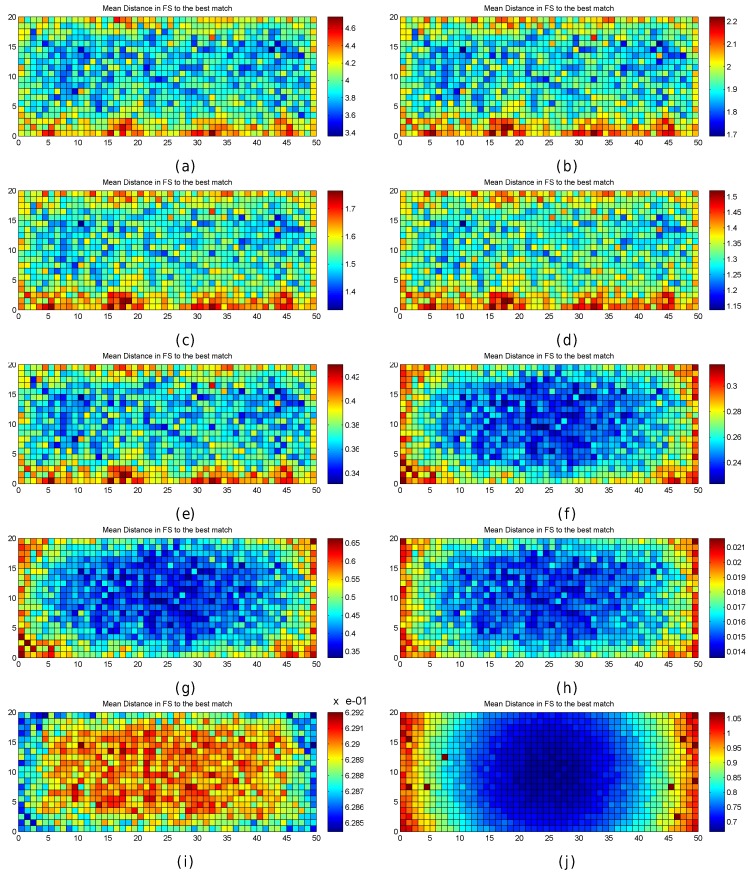
Mean distance in the feature space to the closest match. (**a**) Manhattan distance; (**b**) Euclidean distance; (**c**) Minkowsky3 distance; (**d**) Minkowsky5 distance; (**e**) Mahalanobis distance; (**f**) Matusita distance; (**g**) Neyman distance; (**h**) Sorensen distance; (**i**) Cosine similarity; (**j**) Additive Symmetric distance.

Although the distance in the feature space (RSSI) does not seem to be an indicator of the positioning error, this study goes one step further by showing the mean distance in the feature space to the closest match (calculated with the distance or similarity metric on the RSSI vectors) in each cell of the scenario. Figure 29 shows the mean distance in the feature space to the closest match for the selected metrics except *minkowsky4*, which has been omitted due to its similarity to the other minkowsky metrics (including *Manhattan* and *Euclidean* distances).

According to Figure 29, the 10 selected metrics present three different patterns. Figure 29a–e are similar, and the highest mean distance in the feature space is provided when the operational fingerprints are close to the APs (see Figure 7e–f). Figure 29f–i are similar, and the lowest mean distance in the feature space is provided when the operational fingerprints have a high mean RSSI value (see Figure 7c,d). Although Cosine (Figure 7i presents an inverse pattern, it is a similarity metric (the higher the value, the better), whereas the other ones are distance metrics (the lower the value, the better). Figure 7j presents a different pattern to that of the others, and it seems that the lowest mean distance is provided at the geometric centre of the scenario. It is worth noting that this last distance metric, Additive Symmetric, provides a few distance outliers with values higher than 100 (removed from the figure to improve visibility).

## 5. Relationship between Observed Large Errors and RSSI Values

### 5.1. General Discussion about Sources of Large Errors

This paper has explored the main sources of large errors in deterministic fingerprinting: quantization and noise. Also, the relation between large errors and the assumptions commonly made in some fingerprinting parameters is also explored:

- Quantization of RSSI values has introduced uncertainty to radio signal propagation and a difference of just 1 dBm might correspond to a real difference of ≈0 dBm, 1 dBm or ≈2 dBm. The difference of just 1 dBm means a displacement of a few centimeters or many meters depending on the distance with respect to the antenna. Therefore, the real displacement might be almost 0 (real difference of ≈0 dBm) to twice the expected displacement (real difference of ≈2 dBm).
- The presence of noise has increased uncertainty in radio signal propagation, which makes it more difficult to distinguish relatively close fingerprints.
- Generating the radio map using the grid strategy can be considered a source of error since the underpinning of radio propagation is not considered. Moreover, having a dense grid does not imply low positioning errors since there is a lower bound in the IPS accuracy due to the noise present in RSSI values.
- The AP density and distribution are both important. If the distribution is optimized and the distance between access points is low, the probability of having large errors decreases. However, fingerprinting relies on an already deployed Wi-Fi network in many real cases, which might not be optimized for positioning. Moreover, the presence of virtual APs is usually not exploited. This lack of knowledge about the Wi-Fi network should be considered a source of error, since the AP density (number of APs detected) might not correspond to the expected distribution for proper operation of the IPS (e.g., presence of virtual APs, detection of APs placed in adjacent buildings, among others).
- The fingerprint density, i.e., number of fingerprints per reference point, is also important. On the one hand, relying on just one fingerprint per reference point increases the probability of having large errors since the reference database might contain more fingerprint outliers. On the other hand, having many fingerprints per reference point does not have a significant impact on the accuracy of the IPS (there is a lower bound in the system accuracy), but the computational costs at the operational stage to estimate the position increase. The number of fingerprints have to be carefully balanced to have enough samples to provide good accuracy, keeping the computational costs at the operational level reasonable.
- The reference fingerprints in the realistic world are noisy, therefore there might always be large positioning errors due to outliers at the operational stage. One way to mitigate them is to average reference and operational fingerprints. Averaging fingerprints mitigates the injected noise and the probability of obtaining large errors decreases. Although it can easily be done when generating the radio map (reference fingerprints), averaging at the operational stage is not always possible (it requires the mobile node to be static or having multiple synchronized interfaces).
- The deterministic algorithm used for fingerprinting is not perfect and it should also be considered a source of error. The premise “*the most similar fingerprint in feature space, the closest one in the geometric space*” is not true in most cases (the positioning error is higher than the MEE in more than 90% of cases in the realistic world). Moreover, there is no clear relation between the distance in the feature space and the geometric distance due to the injected noise. This is a fact derived from the quantization and noise present in the RSSI values, but it could also be observed in the optimistic world (no quantization and no noise), where the closest match in the feature space did not correspond to the closest reference fingerprint in the geometric space in ≈2% of cases. The deterministic k-NN is a general-purpose rule that does not consider the nature of radio signal propagation.
- None of the studied distance metrics encompasses the logarithmic nature of radio signal propagation and the location of APs. Therefore, the RSSI values are treated as unitless values and the physical underpinning is ignored. A perfect correlation between a distance/similarity metric and geometric distance was also not obtained in the optimistic world. As already mentioned, the distance metrics might also be a cause of large errors according to [28] since they are general purpose.
- Through this comprehensive study, we observed a pattern: large positioning errors are located in the scenario periphery and far from the APs. In other words, fingerprinting provides good positioning accuracy in those areas that are close to an AP and large errors are not expected. Hopefully, the distance to the closest AP can be calculated from the strongest RSSI value in the fingerprint. This is due to the nature of theoretical radio signal propagation: signal degradation is almost depreciable at long distances.

In the real world, fingerprinting is also affected by external features of the environment and issues in the radio signal propagation. Although they are not within the scope of this paper, we enumerate a few of them:

1. **Intermittent detection of APs:** Distant APs are sometimes detected at a given position; this intermittent effect affects fingerprinting. To partially avoid it, some authors apply thresholds to remove weak signals in the fingerprints.
2. **Device diversity:** This paper has performed a comprehensive study of the sources of large errors with one assumption: the hardware used to collect the fingerprints remains the same for training and operational stages. In the real world, a device with a different antenna gain might be used for positioning. This can also be considered a source of error since the operational fingerprints have a shift in the RSSI values which negatively affects the accuracy [51].
3. **Environmental dynamics:** We have considered a static scenario in our simulations in order to explore the origins of large errors related to the fingerprinting technique. However, one source of large errors is the dynamics of the environment. Positioning accuracy might be highly affected by humidity, differences in the density of people, open/closed doors or in training and operational stages, among other scenario factors [44,52].
4. **Changes in the Wi-Fi network:** Many real Wi-Fi fingerprinting solutions rely on an already deployed Wi-Fi infrastructure for communications. Network configuration is susceptible to changes which might have a catastrophic effect if they are not communicated to the IPS administrators, e.g., replacement of APs, reconfiguration of APs or AP rearranging.

### 5.2. RSSI Values as Indicator of Large Errors

As mentioned in the previous sections, it seems that the simulations showed a pattern in the location of large positioning errors. Most of them were far from the APs, where the maximum RSSI value and mean RSSI values are both low.

**Figure 30:**
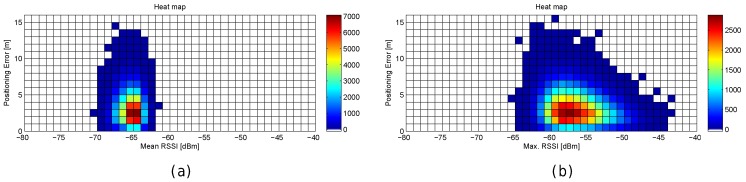
Relation between positioning error and RSSI statistics (mean RSSI (**a**) and maximum RSSI value (**b**)) in the simulated scenario considering the 100 repetitions in the simulation.

In order to conduct an in-depth analysis, the relation between the positioning error and the RSSI statistics (mean and maximum values) was plotted in a scatter plot. Since this kind of plot cannot show the density of tuples with similar values, it was transformed into the heat maps shown in Figure 30. The horizontal axis represents the mean and maximum RSSI value in an operational fingerprint, whereas the vertical axis represents the positioning error by using the 1-NN algorithm. The cell colour indicates how many of the 100,000 analysed fingerprints (1000 operational fingerprints × 100 simulations) fall into this cell.

According to the figures, it seems that the maximum RSSI better represents the upper bound in error positioning in the simulated scenario. On the one hand, the lower the maximum RSSI value, the higher the positioning error that can be obtained. On the other hand, the higher the maximum RSSI value, the more the probability of having a large error decreases. Furthermore, the largest error is lower than 5 m when the max. RSSI is −43 dBm.

**Figure 31:**
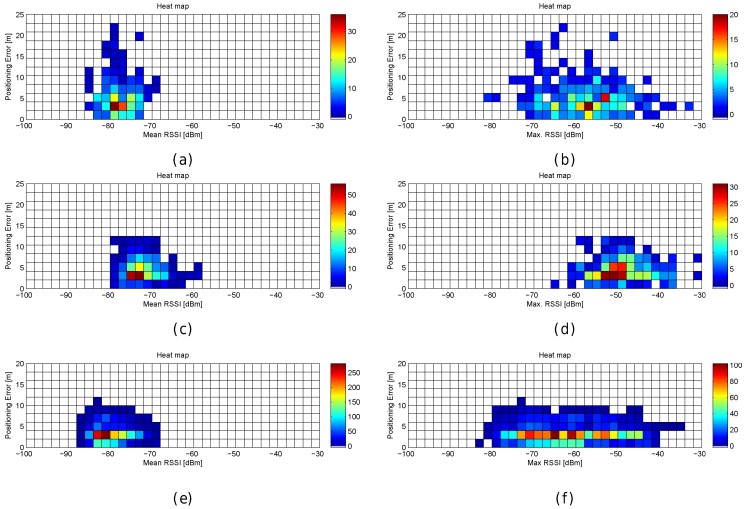
Relation between positioning error and RSSI statistics (mean RSSI and maximum RSSI value) in real scenarios. (**a**,**b**) Departamento de Sistemas de Informação, University of Minho; (**c**,**d**) GEOTEC laboratory, Universitat Jaume I [53] (**e**,**f**) Library, Universitat Jaume I [54].

**Figure 32:**
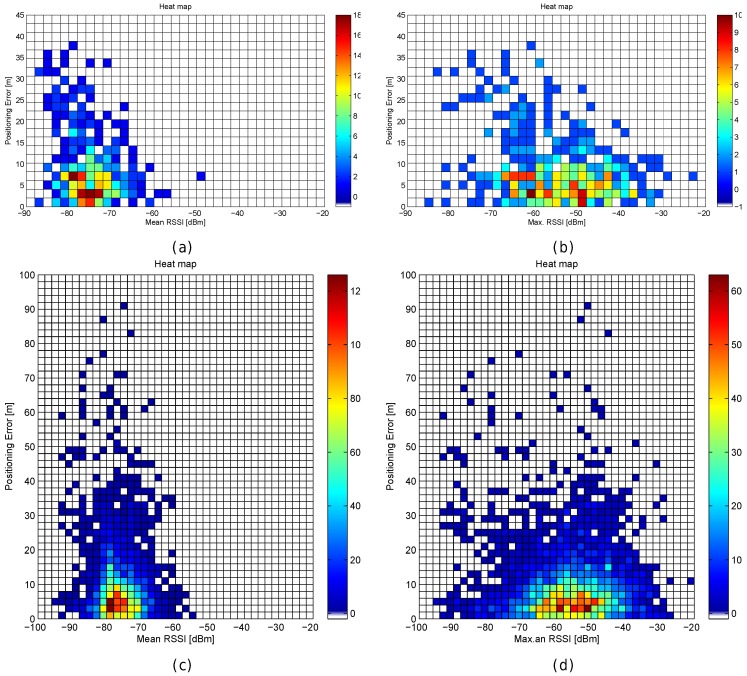
Relation between positioning results in real scenarios and RSSI statistics (mean RSSI and maximum RSSI value). (**a**,**b**) Technical University of Tampere—Building 1 [55,56]; (**c**,**d**) Technical University of Tampere—Tietolato Building [57,58].

Although the simulations mimic realistic scenarios, some additional sources of error that affect the deterministic positioning estimator are not considered (e.g., dynamics in the scenario and other unexpected or “unknown” issues with radio signal propagation). Therefore, to encompass real deployments, an analysis in real world scenarios is shown in Figure 31 and Figure 32. The former figure shows some examples of fingerprinting in real scenarios done by the authors of this paper at their respective institutions. This setup contains one department corridor, one research laboratory [53] and a university library [54] (some details about the datasets and experimental setup can be found in the respective references). The latter figure shows some examples from two external datasets collected by an independent research group [55,56,57,58] at the Technical University of Tampere.

It is worth noting that the network infrastructure is an uncontrolled parameter in all the selected real scenarios since it was already deployed, and it is also an “unknown” parameter since information about its configuration and the exact position of some APs are both unknown. In fact, some APs might be placed in the nearby buildings.

The analysis of the relation between positioning error and RSSI statistics is shown in Figure 31 for our scenarios. First, tests were done using a dataset collected at the Departamento de Sistemas de Informação (University of Minho, PT). Although the number of operational samples is much lower compared to the simulation, the relation between RSSI statistics and positioning error is similar to that observed in the simulation (see Figure 30). Large errors occur when the mean and/or maximum RSSI values are weak. Then, a dataset collected from a research laboratory was used. Although the shapes of both relations are similar to the simulation, the positioning errors are much lower since the size of the environment was small and the density of APs in the scenario was very high (i.e., very large errors did not occur); the results obtained at the university library showed the same behaviour. In general, large errors are not expected when the max. RSSI value is high.

The relation between positioning error and RSSI statistics for the TUT scenarios is shown in Figure 32. The relation between RSSI statistics and positioning error is similar to that observed in the simulation (the stronger the max. and/or mean RSSI, the better the positioning accuracy). However, in these two datasets, the trend is less clear and the positioning error is much larger than in the simulations. It is worth mentioning that only one fingerprint per reference point was collected in both datasets. Moreover, the second dataset was created by means of crowd-sourcing, hence the presence of very large errors, some of them higher than 50 m. Both databases were collected at multi-storey buildings. In general, large errors are not expected when the mean and max. RSSI value is high.

## 6. Conclusions

A comprehensive analysis of the sources of positioning errors has been introduced in this paper. The quantization of RSSI values and the noise present due to “unknown” factors (uncontrolled factors that cannot be easily modelled such as the impact of humidity or the signal diffraction, abortion, reflection and refraction) are the main sources of errors in deterministic fingerprinting. Quantization adds uncertainty to the relation between the RSSI values and the distance to the AP. This uncertainty is low for those areas close to APs but it becomes larger as the distance to the AP increases. The noise present in the environment, due to unknown signal propagation factors, significantly increases the uncertainty. Thus, the use of deterministic fingerprinting is providing a reasonable mean accuracy for many applications where exact precision is not required. However, based on our experience in previous deployments, there are always a few cases where the errors are extremely large.

These large errors motivated us to perform a comprehensive study in which many controlled (depend on the system developer) and uncontrolled (do not depend on the system developer, such as where the APs are placed) parameters of deterministic fingerprinting were analysed: grid size, density and distribution of APs; density of reference and operational fingerprints and k-NN parameters. The results show that many of our assumptions are not true, according to simulated data:

- Having an extremely dense grid does not guarantee extremely good accuracy.
- Adding more APs beyond a threshold has little positive impact on the positioning accuracy, if the APs are uniformly distributed.
- Having hundreds of fingerprints per reference point does not guarantee extremely good accuracy, if data is not appropriately preprocessed.
- The k-NN basic premise when comparing any two fingerprints, “the lowest distance (or highest similarity) in the feature space, the lowest distance in the geometric space” does not hold true in all cases.
- It seems that there is a lower bound in the accuracy by using deterministic fingerprinting according to our simulations. The most important step now is not to provide a better mean positioning error, but to detect and reduce large positioning errors.

The parameters for deterministic fingerprinting are commonly selected according to the average positioning error and do not consider the underpinning nature of signal propagation. To have a better overview of the positioning errors, this study introduced detailed results that showed the accuracy on each grid cell. The graphical results showed that the accuracy of deterministic Wi-Fi fingerprinting positioning depends on the area where the operational fingerprint is located; this paper has depicted a clear pattern: the areas near to the APs report lower positioning errors according to the simulations.

Finally, we checked this in real deployments (no simulation) and the same pattern was detected in all of them. It is worth noting that additional sources of error were introduced in the real scenarios by using arbitrary reference points (no grid), multiple heterogeneous devices which might have different antenna gains, the presence of mobile hotspots, and the presence of reference fingerprints on multiple floors, among many others. Although the selected real scenarios are more complex than the simulated scenario, it can be stated that the probability of having a large error decreases as the maximum value and mean RSSI value in a fingerprint increase, i.e., a fingerprint with a high maximum and mean RSSI value is less prone to providing a large positioning error.

## Figures and Tables

**Table 1:** Examples of how quantization provides a distance difference of two RSSI with ±1 dBm higher than expected.

Dist${}_{1}$	RSSI${}_{1}$	QRSSI${}_{1}$	Dist${}_{2}$	RSSI${}_{2}$	QRSSI${}_{2}$	δRSSI	δQRSSI	δDist [m]	Est. Dist [m]
1.0593	−40.50	−41	1.0594	−40.50	−41	≈0	0	<0.001	0
1.0593	−40.50	−41	1.0592	−40.499	−40	≈0	1	<0.001	≈0.13
1.0593	−40.50	−41	1.1885	−41.499	−41	≈1	0	≈0.13	0
1.0593	−40.50	−41	1.1886	−41.50	−42	≈1	1	≈0.13	≈0.13
1.0592	−40.49	−40	1.1886	−41.50	−42	≈1	2	≈0.13	≈0.26
59.5663	−75.50	−76	59.5664	−75.5	−76	≈0	0	<1 mm	0
59.5663	−75.50	−76	59.5662	−75.499	−75	≈0	1	<1 mm	≈7.27
59.5663	−75.50	−76	66.8343	−76.499	−76	≈1	0	≈7.27	0
59.5663	−75.50	−76	66.8344	−76.50	−77	≈1	1	≈7.27	≈7.27
59.5662	−75.49	−75	66.8344	−76.50	−77	≈1	2	≈7.27	≈14.54
94.4061	−79.50	−80	94.4062	−79.50	−80	≈0	0	<1 mm	0 m
94.4061	−79.50	−80	94.4060	−79.499	−79	≈0	1	<1 mm	≈11.52
94.4061	−79.50	−80	105.9253	−80.499	−80	≈1	0	≈11.52	0
94.4061	−79.50	−80	105.9254	−80.50	−81	≈1	1	≈11.52	≈11.52
94.4060	−79.49	−79	105.9254	−80.50	−81	≈1	2	≈11.52	≈23.04

**Table 2:** Analysis of quantization for Wi-Fi fingerprinting using 1-NN.

	Mean Error	Max. Error	Max. Expected Error	% Cases above the MEE
no quantization	0.389±0.015	0.933±0.009	0.707	1.367
with quantization	0.493±0.027	1.755±0.015	0.707	20.222

**Table 3:** Analysis of grid size for Wi-Fi fingerprinting in the optimistic world using 1-NN: Mean positioning error; Maximum positioning Error; Maximum expected error (MEE) and percentage of cases above the MEE.

Grid	Mean Error	Max. Error	Max. Expected Error	% Cases above the MEE
10.0 m	3.943±0.156	6.292±0.080	7.071	2.003
5.0 m	1.975±0.077	3.607±0.035	3.536	1.875
2.0 m	0.776±0.030	1.600±0.012	1.414	1.416
1.0 m	0.389±0.015	0.933±0.009	0.707	1.367
0.5 m	0.194±0.008	0.525±0.004	0.354	1.340
0.2 m	0.078±0.003	0.227±0.001	0.141	1.296
0.1 m	0.039±0.002	0.119±0.000	0.071	1.279

**Table 4:** Analysis of grid size for Wi-Fi fingerprinting in the quantized world using 1-NN: Mean positioning error; Maximum positioning Error; Maximum expected error (MEE) and percentage of cases above the MEE.

Grid	Mean Error	Max. Error	Max. Expected Error	% Cases above the MEE
10.0 m	3.706±0.157	6.083±0.070	7.071	0.800
5.0 m	1.825±0.079	3.557±0.040	3.536	1.675
2.0 m	0.787±0.039	2.039±0.019	1.414	6.924
1.0 m	0.493±0.027	1.755±0.015	0.707	20.222
0.5 m	0.370±0.021	1.617±0.011	0.354	47.243
0.2 m	0.318±0.019	1.418±0.004	0.141	82.348
0.1 m	0.312±0.017	1.465±0.004	0.071	94.240

**Table 5:** Analysis of grid size for Wi-Fi fingerprinting in the noisy world using 1-NN: Mean positioning error; Maximum positioning Error; Maximum expected error (MEE) and percentage of cases above MEE.

Noise	Grid	Mean Error	Max. Error	Max. Expected Error	% Cases above the MEE
σ=1	10.0 m	4.086±0.180	6.968±0.115	7.071	5.500
	5.0 m	2.389±0.122	5.508±0.101	3.536	17.250
	2.0 m	1.852±0.106	6.121±0.084	1.414	60.872
	1.0 m	1.728±0.099	6.255±0.075	0.707	86.154
	0.5 m	1.647±0.095	6.781±0.054	0.354	95.973
σ=2	10.0 m	4.849±0.245	9.045±0.169	7.071	19.100
	5.0 m	3.761±0.205	9.192±0.156	3.536	49.325
	2.0 m	3.370±0.192	10.870±0.159	1.414	85.744
	1.0 m	3.218±0.184	11.764±0.111	0.707	95.587
	0.5 m	3.102±0.178	12.512±0.098	0.354	98.826
σ=3	10.0 m	5.996±0.328	11.667±0.318	7.071	32.300
	5.0 m	5.254±0.298	13.373±0.238	3.536	68.050
	2.0 m	4.827±0.282	16.322±0.277	1.414	92.312
	1.0 m	4.610±0.268	18.498±0.294	0.707	97.787
	0.5 m	4.492±0.263	21.336±0.412	0.354	99.395
σ=4	10.0 m	7.717±0.425	15.248±0.416	7.071	52.000
	5.0 m	6.826±0.416	18.884±0.569	3.536	79.425
	2.0 m	6.242±0.379	23.570±0.545	1.414	94.924
	1.0 m	6.010±0.365	27.152±0.575	0.707	98.633
	0.5 m	5.855±0.356	32.759±0.656	0.354	99.622
σ=5	10.0 m	9.353±0.567	19.674±0.671	7.071	60.400
	5.0 m	8.308±0.517	23.980±0.655	3.536	83.750
	2.0 m	7.676±0.481	30.767±0.745	1.414	96.768
	1.0 m	7.376±0.468	37.907±0.678	0.707	99.004
	0.5 m	7.191±0.456	43.149±0.509	0.354	99.738

**Table 6:** Location [x,y,z] of the 8 APs.

AP Distribution	AP 1/AP 5	AP 2/AP 6	AP 3/AP 7	AP 4/AP 8
Configuration 1	[625,500,390]	[1875,500,390]	[3150,500,390]	[4375,500,390]
	[625,1500,540]	[1875,1500,540]	[3150,1500,540]	[4375,1500,540]
Configuration 2	[0,0,390]	[1666,666,390]	[3333,666,390]	[5000,0,390]
	[000,2000,540]	[1666,1333,540]	[3333,1333,540]	[5000,2000,540]
Configuration 3	[000,666,390]	[1666,0,390]	[3333,0,390]	[5000,666,390]
	[000,1333,540]	[1666,2000,540]	[3333,2000,540]	[5000,1333,540]
Configuration 4	[500,100,390]	[1750,100,390]	[3250,100,390]	[4500,100,390]
	[500,1900,540]	[1750,1900,540]	[3250,1900,540]	[4500,1900,540]
Configuration 5	[313,1000,390]	[938,1000,390]	[1563,1000,390]	[2188,1000,390]
	[2813,1000,390]	[3438,1000,390]	[4063,1000,390]	[4688,1000,390]

**Table 7:** Analysis of AP distribution for Wi-Fi fingerprinting using 1-NN.

AP Dist	Mean Error	Max. Error	Max. Expected Error	% Cases above the MEE
Orig	3.237±1.860	12.560	0.707	95.540
Alt 1	3.169±1.962	18.609	0.707	95.050
Alt 2	3.580±2.364	20.622	0.707	95.850
Alt 3	3.433±2.059	17.337	0.707	95.900
Alt 4	3.251±1.868	14.333	0.707	95.470
Alt 5	6.140±5.134	23.195	0.707	96.790

**Table 8:** Analysis of virtual APs for Wi-Fi fingerprinting in the realistic world using 1-NN.

AP Dist	Independent	Mean Error	Max. Error	Max. Expected Error	% Cases above the MEE
1 × 8 AP	-	3.218±0.184	11.764±0.111	0.707	95.587
1 × 32 AP	-	1.967±0.102	6.753±0.078	0.707	89.703
4 × 8 AP	yes	2.160±0.121	7.495±0.067	0.707	91.146
avg × 8 AP	yes	1.815±0.102	6.517±0.065	0.707	87.636
4 × 8 AP	partial	2.635±0.148	9.321±0.083	0.707	93.794
avg × 8 AP	partial	2.420±0.137	8.653±0.092	0.707	92.836
4 × 8 AP	no	3.206±0.184	11.710±0.157	0.707	95.640
avg × 8 AP	no	3.209±0.183	11.569±0.114	0.707	95.733

**Table 9:** Analysis of averaging training and operational fingerprints in the noisy world using 1-NN.

# Ref. FP	Ref. avg.	Op. avg.	Mean Error	Max. Error	MEE	% Cases above the MEE
1	no avg	no avg	3.455±1.969	12.096±1.140	0.707	96.284
10	no avg	no avg	3.216±1.837	11.692±1.221	0.707	95.754
10	no avg	5	2.454±1.422	8.797±0.860	0.707	92.606
10	no avg	10	2.344±1.376	8.509±0.877	0.707	91.922
10	5	no avg	2.774±1.605	10.446±1.110	0.707	94.051
10	5	5	1.687±0.960	6.152±0.722	0.707	85.724
10	10	no avg	2.753±1.598	10.520±1.141	0.707	94.043
10	10	10	1.336±0.762	4.865±0.548	0.707	78.268
100	5	5	1.534±0.877	5.649±0.602	0.707	83.034
100	10	10	1.168±0.672	4.338±0.429	0.707	72.644

**Table 10:** Analysis of the k-Value for Wi-Fi fingerprinting in the noisy world using k-NN: Mean positioning error; Maximum positioning Error; Maximum expected error (MEE) and percentage of cases above the MEE.

*k*-Value	Mean Error	Max. Error	Max. Expected Error	% Cases above the MEE
1	3.218±0.184	11.764±0.111	0.707	95.587
3	2.619±0.146	9.297±0.101	0.707	94.088
5	2.498±0.138	8.753±0.092	0.707	93.485
7	2.442±0.134	8.401±0.079	0.707	93.329
9	2.401±0.132	8.420±0.078	0.707	93.049
11	2.383±0.131	8.394±0.099	0.707	93.120
13	2.377±0.130	8.427±0.101	0.707	93.048
15	2.364±0.129	8.438±0.091	0.707	92.934
17	2.350±0.128	8.101±0.097	0.707	92.928
19	2.352±0.128	8.096±0.084	0.707	92.990
21	2.348±0.128	8.114±0.091	0.707	93.032
23	2.348±0.128	8.064±0.083	0.707	92.917
25	2.342±0.127	8.267±0.102	0.707	92.921
27	2.336±0.127	8.141±0.086	0.707	93.016
29	2.337±0.126	8.069±0.087	0.707	92.994
31	2.335±0.126	7.990±0.072	0.707	93.049
33	2.340±0.126	7.956±0.079	0.707	93.044
35	2.332±0.126	8.150±0.099	0.707	93.068
37	2.337±0.126	8.059±0.111	0.707	92.938
39	2.334±0.126	8.036±0.084	0.707	92.975
41	2.328±0.126	7.942±0.085	0.707	92.948
43	2.337±0.126	7.936±0.083	0.707	92.952
45	2.334±0.126	8.173±0.100	0.707	92.897
47	2.330±0.125	8.124±0.093	0.707	92.924
49	2.333±0.125	7.940±0.085	0.707	93.080
51	2.333±0.125	7.904±0.076	0.707	93.067
53	2.338±0.125	7.981±0.088	0.707	93.024
55	2.332±0.125	8.057±0.091	0.707	93.083
57	2.337±0.125	8.043±0.110	0.707	93.021
59	2.333±0.125	7.924±0.085	0.707	92.955
61	2.330±0.125	7.903±0.088	0.707	93.005
63	2.332±0.125	7.948±0.101	0.707	93.026
65	2.336±0.125	7.992±0.090	0.707	93.082
67	2.336±0.125	8.086±0.090	0.707	93.083
69	2.333±0.125	7.941±0.082	0.707	92.994
71	2.335±0.125	7.961±0.088	0.707	93.026
73	2.339±0.125	7.900±0.071	0.707	93.047
75	2.339±0.125	7.918±0.092	0.707	93.153

**Table 11:** Analysis of similarity/distance metrics for Wi-Fi fingerprinting in the optimistic and quantized worlds using 1-NN.

Metric	Optimistic				Quantized			
	MeanPE	MaxPE	%	Corr.	MeanPE	MaxPE	%	Corr.
additivesymmetric	1.424±0.054	2.526	92.373	0.502	0.759±0.045	2.422	79.517	0.545
bhattacharyya	7.410±0.456	17.003	99.090	0.953	7.914±0.480	18.493	98.637	0.942
camberra	0.407±0.018	1.453	11.681	0.748	0.580±0.032	2.009	55.760	0.356
chebyshev	0.412±0.019	1.166	15.922	0.748	0.499±0.028	1.712	70.725	0.099
cityblock	0.389±0.015	0.943	6.913	0.872	0.493±0.027	1.783	60.349	0.066
cityblockintersect	14.136±0.647	26.610	99.887	NaN	14.133±0.647	26.590	99.894	NaN
clarck	0.632±0.221	40.854	29.657	0.561	0.609±0.036	2.529	57.973	0.441
cosine	0.395±0.016	1.143	9.236	0.701	0.572±0.033	3.020	54.094	0.301
divergence	0.415±0.020	1.658	12.735	0.210	0.598±0.034	2.255	57.303	0.425
euclidean	0.392±0.016	1.029	8.536	0.854	0.492±0.027	1.783	60.362	0.073
fidelity	7.410±0.456	17.003	99.090	0.959	7.914±0.480	18.493	98.637	0.947
harmonicmean	4.980±0.282	11.044	97.962	0.885	5.232±0.289	13.668	97.638	0.831
hellinger	0.388±0.015	1.017	5.999	0.913	0.578±0.032	2.096	55.643	0.366
innerproduct	5.396±0.271	13.862	98.959	0.426	5.850±0.308	14.879	99.006	0.316
jeffreys	0.388±0.015	1.020	6.005	0.874	0.578±0.032	2.096	55.646	0.346
jensen-difference	0.388±0.015	1.017	5.998	0.875	0.578±0.032	2.096	55.643	0.348
jensen-shannon	3.631±0.190	7.988	96.219	−0.873	3.780±0.197	9.551	97.181	−0.764
k-divergence	13.722±0.631	25.780	99.892	−0.229	13.212±0.660	25.780	99.802	−0.431
kullback-leibler	12.064±0.627	24.570	99.655	−0.035	12.356±0.665	24.873	99.456	−0.083
kumar-hassebrook	0.392±0.016	1.019	8.567	0.817	0.568±0.031	1.968	53.938	0.338
KumarJohnson	0.388±0.015	1.019	5.998	0.875	0.578±0.032	2.096	55.662	0.350
lorentzian	0.389±0.015	0.934	6.794	0.873	0.494±0.027	1.788	60.367	0.072
mahalanobis	0.391±0.015	0.974	8.308	0.853	0.554±0.030	1.937	54.514	0.287
matusita	0.388±0.015	1.017	5.999	0.913	0.578±0.032	2.096	55.643	0.366
MaxSymetricChi2	0.389±0.015	1.030	6.083	0.863	0.579±0.032	2.097	55.756	0.350
minkowsky3	0.397±0.016	1.093	10.420	0.827	0.492±0.027	1.783	60.362	0.075
minkowsky4	0.401±0.017	1.111	11.771	0.809	0.492±0.027	1.783	60.362	0.076
minkowsky5	0.404±0.018	1.124	12.717	0.796	0.492±0.027	1.783	60.362	0.077
MinSymetricChi2	0.388±0.015	0.999	5.920	0.877	0.574±0.032	2.033	55.544	0.317
motyka	0.389±0.015	0.940	6.931	0.891	0.525±0.029	1.796	57.392	0.217
neyman	0.389±0.015	1.021	6.143	0.875	0.578±0.032	2.033	55.224	0.331
pearson	0.388±0.015	1.015	5.926	0.874	0.573±0.031	2.037	55.478	0.325
sorensen	0.389±0.015	0.940	6.931	0.891	0.525±0.029	1.796	57.392	0.217
squared	0.388±0.015	1.015	5.993	0.874	0.578±0.032	2.096	55.641	0.348
squaredchord	0.388±0.015	1.017	5.999	0.874	0.578±0.032	2.096	55.643	0.348
Taneja	0.388±0.015	1.021	6.009	0.874	0.578±0.032	2.096	55.616	0.346
topsoe	0.388±0.015	1.017	5.998	0.875	0.578±0.032	2.096	55.643	0.348
VicisSymetricChi2A	0.415±0.020	1.664	12.811	0.133	0.598±0.034	2.255	57.321	0.414
VicisSymetricChi2B	0.389±0.015	1.026	6.058	0.864	0.579±0.032	2.096	55.721	0.348
VicisSymetricChi2C	0.388±0.015	1.003	5.948	0.877	0.575±0.032	2.031	55.535	0.323
VicisWaveHedges	0.408±0.019	1.492	11.753	0.656	0.581±0.032	2.009	55.875	0.360
wavehedges	0.407±0.018	1.430	11.607	0.757	0.576±0.032	1.921	55.637	0.329

**Table 12:** Analysis of similarity/distance metrics for Wi-Fi fingerprinting in the realistic world using 1-NN.

Metric	Optimistic			
	MeanPE	MaxPE	%	Corr.
additivesymmetric	3.594±0.200	12.534	97.790	0.016
cityblock	3.199±0.184	11.642	98.170	−0.054
cosine	3.588±0.205	12.672	97.829	0.024
euclidean	3.312±0.189	11.676	97.756	−0.016
mahalanobis	3.404±0.193	11.729	97.505	−0.005
matusita	3.469±0.196	12.153	97.641	0.023
minkowsky3	3.344±0.191	11.840	97.700	−0.007
minkowsky4	3.347±0.191	11.850	97.713	−0.004
minkowsky5	3.348±0.191	11.847	97.716	−0.005
neyman	3.462±0.195	12.119	97.621	0.014
sorensen	3.391±0.191	12.083	97.692	0.004

## Abbreviations

The following abbreviations are used in this manuscript:

AP	Access Point
CDF	Cumulative Distribution Function
FS	Feature Space
FSPL	Free Space Path Loss
GNSS	Global Navigation Satellite System
IPS	Indoor Positioning System
kNN	k Nearest Neighbors
LBS	Location Based Services
MEE	Maximum Expected Error
IPIN	Indoor Positioning and Indoor Navigation
RFID	Radio-Frequency Identification
RSSI	Received Signal Strength Indicator
RTLS	Real Time Location System
